# Interplay of early negative life events, development of orbitofrontal cortical thickness and depression in young adulthood

**DOI:** 10.1002/jcv2.12210

**Published:** 2023-12-06

**Authors:** Lea L. Backhausen, Jonas Granzow, Juliane H. Fröhner, Eric Artiges, Marie‐Laure Paillère‐Martinot, Hervé Lemaître, Fabio Sticca, Tobias Banaschewski, Sylvane Desrivières, Antoine Grigis, Andreas Heinz, Rüdiger Brühl, Dimitri Papadopoulos‐Orfanos, Luise Poustka, Sarah Hohmann, Lauren Robinson, Henrik Walter, Jeanne Winterer, Gunter Schumann, Jean‐Luc Martinot, Michael N. Smolka, Nora C. Vetter, the IMAGEN Consortium

**Affiliations:** ^1^ Department of Psychiatry and Psychotherapy TUD Dresden University of Technology Dresden Germany; ^2^ Department of Child and Adolescent Psychiatry Medical Faculty and University Hospital Carl Gustav Carus, TUD Dresden University of Technology Dresden Germany; ^3^ Institut National de la Santé et de la Recherche Médicale INSERM U1299 “Trajectoires développementales en psychiatrie” Université Paris‐Saclay Ecole Normale supérieure Paris‐Saclay CNRS Centre Borelli Gif‐sur‐Yvette France; ^4^ Department of Psychiatry Lab‐D‐Psy EPS Barthélémy Durand Etampes France; ^5^ Department of Child and Adolescent Psychiatry Pitié‐Salpêtrière Hospital Paris France; ^6^ NeuroSpin CEA Université Paris‐Saclay Gif‐sur‐Yvette France; ^7^ Institute for Educational Support for Behaviour, Social‐Emotional, and Psychomotor Development University of Teacher Education in Special Needs Zurich Switzerland; ^8^ Department of Child and Adolescent Psychiatry and Psychotherapy Central Institute of Mental Health Medical Faculty Mannheim Heidelberg University Mannheim Germany; ^9^ Centre for Population Neuroscience and Precision Medicine (PONS) Institute of Psychiatry, Psychology & Neuroscience SGDP Centre King's College London London UK; ^10^ Department of Psychiatry and Neurosciences Charité – Universitätsmedizin Berlin Corporate Member of Freie Universität Berlin Humboldt‐Universität zu Berlin, and Berlin Institute of Health Berlin Germany; ^11^ Physikalisch‐Technische Bundesanstalt (PTB) Braunschweig and Berlin Berlin Germany; ^12^ Department of Child and Adolescent Psychiatry and Psychotherapy University Medical Centre Göttingen Göttingen Germany; ^13^ Department of Child and Adolescent Psychiatry Psychotherapy and Psychosomatics University Medical Center Hamburg Eppendorf Hamburg Germany; ^14^ Department of Psychological Medicine Section for Eating Disorders Institute of Psychiatry, Psychology and Neuroscience King's College London London UK; ^15^ Department of Education and Psychology Freie Universität Berlin Berlin Germany; ^16^ Department of Psychiatry and Psychotherapy PONS Research Group Campus Charite Mitte Humboldt University Berlin and Leibniz Institute for Neurobiology Magdeburg Germany; ^17^ Institute for Science and Technology of Brain‐inspired Intelligence (ISTBI) Fudan University Shanghai China; ^18^ Department of Psychology MSB Medical School Berlin Berlin Germany

**Keywords:** adolescence, depression, life events, longitudinal studies, structural MRI (sMRI)

## Abstract

**Background:**

Early negative life events (NLE) have long‐lasting influences on neurodevelopment and psychopathology. Reduced orbitofrontal cortex (OFC) thickness was frequently associated with NLE and depressive symptoms. OFC thinning might mediate the effect of NLE on depressive symptoms, although few longitudinal studies exist. Using a complete longitudinal design with four time points, we examined whether NLE during childhood and early adolescence predict depressive symptoms in young adulthood through accelerated OFC thinning across adolescence.

**Methods:**

We acquired structural MRI from 321 participants at two sites across four time points from ages 14 to 22. We measured NLE with the Life Events Questionnaire at the first time point and depressive symptoms with the Center for Epidemiologic Studies Depression Scale at the fourth time point. Modeling latent growth curves, we tested whether OFC thinning mediates the effect of NLE on depressive symptoms.

**Results:**

A higher burden of NLE, a thicker OFC at the age of 14, and an accelerated OFC thinning across adolescence predicted young adults' depressive symptoms. We did not identify an effect of NLE on OFC thickness nor OFC thickness mediating effects of NLE on depressive symptoms.

**Conclusions:**

Using a complete longitudinal design with four waves, we show that NLE in childhood and early adolescence predict depressive symptoms in the long term. Results indicate that an accelerated OFC thinning may precede depressive symptoms. Assessment of early additionally to acute NLEs and neurodevelopment may be warranted in clinical settings to identify risk factors for depression.

## Graphical abstract

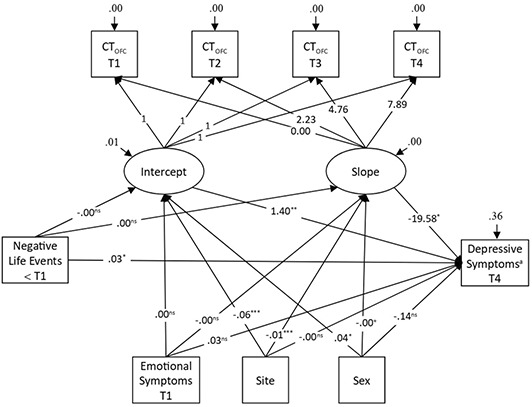

**Key points**

- Severe forms of early negativ life events (NLE) are cross‐sectionally associated both with alterations in the orbitofrontal cortex (OFC) and depressive symptoms in adulthood.
- This study is the first to examine early moderate NLE in childhood predict depressive symptoms in adulthood through accelerated OFC thinning.
- In a complete longitudinal design with four imaging exams, depressive symptoms in young adulthood were predicted by more childhood NLE, a thicker OFC at age 14 and an accelerated OFC thinning across adolescence. OFC development during adoelscence did not mediate effects of NLE on depressive symptoms.
- The assessment of early NLE in addition to acute NLE may be warranted in clinical practice to identify individuals at risk for depression.

## INTRODUCTION

Childhood and adolescent negative life events (early NLE) can influence structural brain development and may thus act as long‐term risk factors for psychopathology like major depressive disorder (MDD) in adulthood (Li et al., 2016).

Previous research focused on childhood adversity such as abuse and deprivation that require significant adaption and represent a deviation from the expectable environment (McLaughlin, 2016). Here, we define early NLE as unpleasantly perceived events that may exceed an individual's regulatory coping resources (Pechtel & Pizzagalli, 2011), like accidents, illness and death, problems with family, friends and sexuality, or social and academic problems (Newcomb et al., 1981). As these events have been rarely studied (e.g. Bartlett et al., 2019), we aimed at shedding light on their impact on neurodevelopment and psychopathology in adulthood.

Severe forms of early NLE have been associated with volume alterations in the orbitofrontal cortex (Ansell et al., 2012; De Brito et al., 2013; Holz et al., 2015). Cortical volume can be further disentangled as a product of cortical thickness (CT) and surface area (SA; Backhausen et al., 2021), yet only one study examined early NLE's effects on CT and SA (Monninger et al., 2020). Changes across adolescence into young adulthood in cortical volume are mainly due to cortical thickness, that is, cortical thinning, rather than changes in SA (Tamnes et al., 2017; Wierenga et al., 2014). Therefore, to examine early NLE's effects on brain development from adolescence into young adulthood, we targeted the development of OFC thickness.

Reduced OFC thickness has been observed in adolescents exposed to childhood adversity versus those without this experience (Gold et al., 2016; Lim et al., 2018). Currently, only cross‐sectional studies demonstrated this, so our longitudinal MRI approach across adolescence into young adulthood fills a research gap.

To assess effects of early NLE on OFC thickness it is essential to take a closer look at the role of the OFC. Emotional stimuli activate the OFC. Its damage alters the experience of negative and positive emotions linking it to psychopathology such as depressive symptoms (Hornak, 2003; Rolls et al., 2020). As part of the prefrontal cortex, the OFC is one of the last regions to fully develop (Arain et al., 2013). Given its extended maturation into the twenties, the OFC might be particularly sensitive to early NLE. From a developmental perspective, cross‐sectionally observed reduced OFC thickness in response to NLE could be based on accelerated maturation, that is, thinning of the OFC across adolescence. This accelerated maturation in response to early adversity is postulated by the Stress Acceleration Hypothesis, specifically in brain structures relevant for emotional learning and reactivity mirroring an early system adaptation to meet immediate emotional demands (Callaghan & Tottenham, 2016). To get a better insight into these relationships, longitudinal investigations, are mandatory.

OFC alterations have also been consistently found in adults with MDD (Arnone et al., 2012; Kempton et al., 2011). Therefore, the OFC might mediate the effects of early NLE on depressive symptoms in young adults. Evidence is provided by reduced medial OFC thickness in adults with MDD in a recent mega‐analysis (Schmaal et al., 2020). To test whether longitudinally accelerated OFC thinning precedes reduced thickness in adults with MDD, the OFC development in adolescence needs to be investigated.

There are only two longitudinal studies examining the relation between OFC thinning and depressive symptoms assessing participants with up to three time points from childhood to adulthood (Bos et al., 2018; Ducharme et al., 2014) with conflicting results. Participants from age nine to 20 with mild to severe depressive symptoms four years later showed accelerated frontal thinning in precentral regions and the lateral OFC (Bos et al., 2018). In contrast, a slower rate of cortical thinning in the medial OFC was found in participants aged five to 18 years with high anxious/depressed symptoms (Ducharme et al., 2014). Diverging findings could stem from different study designs, age ranges studied, measurement instruments for depressive symptomatology, and types of statistical analyses. While Bos et al. (2018) investigated the influence of cortical thinning on depressive symptoms, Ducharme et al. (2014) evaluated the cross‐sectional correlation between anxious/depressive scores and cortical thickness at each visit. Notably, since both studies used accelerated longitudinal designs with up to three time points, a broad age range from mid‐childhood to adulthood was examined, while individual trajectories could only be followed for 6 years reducing the number of data sets in a given age span.

To our knowledge, there is currently only one recent study investigating the whole sequence of early NLE effects on OFC thickness and depressive symptoms (Monninger et al., 2020). A heightened exposure to NLE in early childhood was associated with reduced thickness in the right OFC and elevated depressive symptoms at the age of 25 (Monninger et al., 2020). While this study comprised several assessments of NLE, OFC thickness was measured only cross‐sectionally in early adulthood. Consequently, effects of early NLE on longitudinal trajectories of OFC thinning across adolescence remain inconclusive.

To sum up, previous studies suggest that accelerated OFC thinning across adolescence may mediate long‐term effects of early NLE on depressive symptoms in young adulthood. To our knowledge, there are yet no longitudinal studies directly investigating these relationships. Importantly a longitudinal design is crucial to reveal the temporal order of these three core factors (i.e. is a thinner OFC a consequence of or a risk factor for depressive symptoms).

Therefore, we examined their association in a complete longitudinal design spanning four waves of structural MRI data from 321 typically developing adolescents from two sites of the imaging genetics (IMAGEN) project (Schumann et al., 2010). Here, all participants started at the same age (14 years) and were followed across the whole age‐range of interest (at ages 16, 19 and 22). For the first time, we tested a latent growth curve model (LGCM) with OFC thickness as a potential mediator between early NLE and depressive symptoms in young adulthood. First, based on the Stress Acceleration Hypothesis, we expect that a higher burden of early NLE would lead to an accelerated OFC thinning across adolescence (Callaghan & Tottenham, 2016). Second, we hypothesized that an accelerated OFC thinning across adolescence would predict depressive symptoms in young adulthood (Bos et al., 2018). Third, on a psychopathological level, we expected to replicate that a higher burden of early NLE would predict heightened depressive symptoms in young adulthood (Heim & Binder, 2012). Bringing together the individual hypotheses, we expected this effect to be mediated by an accelerated OFC thinning across adolescence.

## METHODS AND MATERIALS

### Participants

Originally, we assessed 534 adolescents (275 male participants) from two different sites (Site 1: *n* = 260; Site 2: *n* = 274) as part of the IMAGEN project (for details on recruitment and assessment procedures see Schumann et al. (2010)). Only these IMAGEN sites provide four MRI time points (additionally at age 16). During their first assessment, participants were about 14 years old with follow‐up assessments at ages 16, 19, and 22. In the original sample, there was missing data due to drop‐outs and non‐completed questionnaires. Therefore, the final sample consisted of 321 participants (175 male participants) of whom all provided data of NLE at their first and depressive symptoms at their last assessment and had adequate‐quality structural MRI data for at least one time point. For details on demographics of the final sample, see Table 1 and Table S2 separated by site. Figure S1 shows the age and sex distribution per time point for each site. Of the 321 participants included in the analyses 94.7% were White (Site 1: 98.1%; Site 2: 91.3%) and the majority stemmed from rather well‐educated households (as a proxy for socioeconomic status). Of 41.1% of participants, both parents and of 77.3%, at least one parent had a university or college degree (university of applied sciences). Exclusion criteria at the first time point included existing bipolar disorder, schizophrenia, and major neuro‐developmental disorders such as autism, as well as a premature birth, head trauma and history of severe neurological or medical disorders. A detailed characterization of the sample in terms of psychopathology can be found in Table S9 for age 14 (using the Development and Well‐Being Assessment; Goodman et al., 2000 at both sites), Table S10 for age 22 at site 1 (using The Structured Clinical Interview for DSM‐IV; Wittchen et al., 1997), and Table S11 for age 22 at site 2 (using the Mini‐International Neuropsychiatric Interview; Sheehan et al., 1998).

**TABLE 1:** Sample characteristics including demographics.

Characteristic	*n*	%
Sex (female)	175	54.52
Handedness (right‐handed)^a^	283	88.71
Non‐white ethnicity	17	5.3

	Mean	SD	Range (min – max)
Age (T1), years	14.44	0.43	13.33–15.61
Age (T2), years	16.68	0.52	15.65–17.88
Age (T3), years	19.2	0.81	17.26–21.91
Age (T4), years	22.33	0.67	20.1–24.66
Height, cm	167.5	7.67	150–186
Weight, kg	57.15	10.71	32–110
BMI	20.28	2.95	14.22–35.51
IQ^b^	112.84	9.9	81.5–141.5

	Median	MAD	Range (min – max)
Pubertal status^c^	4	0	1 ‐ 5
Alcohol use, AUDIT	0	0	0–9
Emotional symptoms, SDQ	2	2.97	0–9
Negative life events, LEQ	6	2.97	0–24
Depressive symptoms (T4), CES‐D	9	7.41	0–49

*Note*: Unless otherwise stated, statistics are given for the first time point. We report data for all participants in the final data set independent of a high‐quality scan at the first time point. Please note that sociodemographic information was missing for few participants (handedness: *n* = 2; ethnicity: *n* = 1; weight, height and BMI: *n* = 4, IQ: *n* = 10; pubertal status: *n* = 1; alcohol use: *n* = 2). The percentages indicated refer to the available information.

Abbreviations: AUDIT, Alcohol Use Disorders Identification Test (Saunders et al., 1993); BMI, body mass index; CES‐D, Center for Epidemiologic Studies Depression Scale (Radloff, 1977); LEQ, Life Event Questionnaire (Newcomb et al., 1981); SDQ, Strengths and Difficulties Questionnaire (Goodman, 1997); T1‐T4, first (baseline) to fourth time point.

^a^ Remaining participants reported left‐handedness except for two participants who reported ambidextrous‐handedness.

^b^ General cognitive ability estimated with the subtests Similarities, Block Design, Vocabulary, and Matrices from the Wechsler Intelligence Scale for Children Fourth Edition (WISC®‐IV; Wechsler, D., 2003).

^c^ Pubertal status ranged from 1 for ‘prepubertal’ to 5 for ‘postpubertal status’, measured with the Pubertal Development Scale (PDS; Petersen et al., 1988) with the value 4 corresponding to ‘advanced pubertal’.

### Negative life events

During the first assessment, participants completed an adapted version of the Life Events Questionnaire (LEQ) covering 39 life events which typically occur during childhood and adolescence (Newcomb et al., 1981; see Table S4). Participants indicated first, whether they had ever experienced an event (i.e. ever in their lifetime) and second, rated how they would feel in consequence of the event, with the response options ranging from “very unhappy to “very happy”. We only included events in the total score if participants had (1) experienced them and (2) rated them as “unhappy” or “very unhappy”. Events rated as “very unhappy” were included in the overall score with double weighting (following an approach of Swartz et al., 2014), resulting in a score from 0 to 78.

### Depressive symptoms

During the fourth assessment, participants rated depressive symptoms with the Center for Epidemiologic Studies Depression Scale (CES‐D; Radloff, 1977). Participants were asked to rate the frequency of 20 items concerning depressive symptoms within the last week. Response options ranged from “Rarely or Never” (0), “Some or a Little Amount of Time” (1), “Occasionally or a Moderate Amount of Time” (2), “Most or All of the Time” (3), resulting in a score from 0 to 60.

### Structural magnetic resonance image acquisition and processing

Structural T1‐weighted magnetic resonance images were acquired during each assessment using a Siemens Trio 3T whole‐body MR tomograph (MAGNETOM Trio, Siemens, Erlangen, Germany) equipped with a 12‐channel headcoil during the first two time points and a 32‐channel headcoil at time points three and four in Site 1. In Site 2, T1‐weighted magnetic resonance images were acquired using Siemens Trio 3T whole‐body MR tomographs (MAGNETOM Trio, Siemens, Erlangen, Germany) equipped with a 12‐channel headcoil during the first two assessments. A different scanner of the same type was used during each of these two assessments. During the third assessment, the Siemens Trio tomograph used during the second assessment in Site 2 received an update to Siemens Prisma 3T whole‐body MR tomograph (MAGNETOM Prisma, Siemens, Erlangen, Germany), which was used equipped with a 64‐channel headcoil subsequently during the third and fourth assessment. High‐resolution T1‐weighted images were collected using a magnetization prepared rapid acquisition gradient‐echo (MPRAGE) sequence [Site 1/Site 2: repetition time (TR) = 1900/2300 ms, echo time (TE) = 2.26/2.93 ms, inversion time (TI) = 900/900 ms, voxel size = 1.0 × 1.0 × 1.0/1.1 × 1.1 × 1.1 mm, flip angle = 9/9°; matrix size = 256 × 256/256 × 256 mm; 176/160 slices]. This information is summed up in Table S8. Both sites regularly performed a standardized scanner quality control (phantom and in‐vivo scan, see Schumann et al. (2010). All images were examined by a clinical neuroradiologist for structural abnormalities.

Image processing was conducted using the longitudinal pipeline of FreeSurfer 6.0.0 (http://surfer.nmr.mgh.harvard.edu; Fischl et al., 2002; Reuter et al., 2012). The recon‐all‐flag ‐3T was used which alters FreeSurfer's internal N3 bias field correction parameters (Sled et al., 1998). CT, SA, and subcortical volume estimates were computed for the Desikan‐Killiany atlas regions. In this study, we selected the CT in the OFC a priori as the measure of interest. Previous study results provide evidence for both right‐lateral (Ducharme et al., 2014; Gold et al., 2016; Lim et al., 2018) and bilateral (Ansell et al., 2012; Bos et al., 2018; De Brito et al., 2013) changes in OFC in relation to NLE and depressive symptoms. As a recent mega‐analysis comparing 1905 patients with major depressive disorder with 7658 healthy individuals reported reduced bilateral OFC thickness (Schmaal et al., 2020), we refrained from considering left and right OFC thickness separately in our analyses. The calculation of mean CT in the OFC is based on the following formula, according to recommendations on the FreeSurferWiki: *mean CT* = ((left CT* left SA) + (right CT * right SA))/(left SA + right SA) (please see https://surfer.nmr.mgh.harvard.edu/fswiki/UserContributions/FAQ). We made no manual adjustments of CT. For outliers in OFC thickness in each time point indicated by boxplots, we thoroughly inspected FreeSurfer outputs and confirmed that the deviations were physiologically plausible and not due to imaging or processing artifacts. No datasets were excluded due to outliers.

For quality assurance, one of three trained operators performed a visual preliminary quality control on each raw T1‐weighted image according to a workflow described by Backhausen et al. (2016). For raw images with questionable image quality, we visually inspected the corresponding longitudinally processed images for accuracy of cortical parcellation following examples from the ENIGMA Cortical Quality Control Guide 2.0, available at https://enigma.ini.usc.edu/protocols/imaging‐protocols/. In addition, we performed post‐processing quality control on images which were flagged by Qoala‐T (Klapwijk et al., 2019). Qoala‐T is a supervised‐learning tool for quality control of FreeSurfer segmented MRI data and is based on FreeSurfer outputs, that is, after passing the automated processing pipeline. Flagged images were either deemed decent and included or poor and thus excluded from further analyes.

Of the original 1494 images available, 177 images (11.85%) were excluded during quality control. As 176 participants, who had a total of 331 high‐quality scans, had missing questionnaires (either LEQ or CES‐D) and could not be included in further analyses, the final sample consisted of 986 images. Details on the quality control flow and exclusion of participants are provided in Table S1, available online.

### Statistical analysis

To test whether early NLE predict depressive symptoms in young adulthood via alterations in OFC thinning, we proceeded in two steps: In the first step, we constructed and compared multiple unconditional LGCM (i.e., without covariates): An intercept‐only model, linear slope models with homo‐ and heteroscedastic residual error structure and a quadratic slope model. The intercept was modeled as the OFC thickness at the first time point by setting all loadings to 1. The slope was modeled as linear change of OFC thickness between the four measurements by constraining the loadings to 0, 2.23, 4.76, and 7.89 (i.e., mean distances between repeated measurements and the first assessment in years). For the quadratic slope model, we additionally modelled a quadratic factor with loadings constrained to the square of linear slope loadings. Across these models, we evaluated overall model fit using the standard criteria (Hu & Bentler, 1999): TLI ≥0.95, CFI ≥0.95, RMSEA ≤0.06, *χ* ^2^/df < 3.00. We sought a best‐fit model based on likelihood ratio tests to evaluate the difference between nested models. In the second step, we constructed a multiple‐mediators model by introducing depressive symptoms and early NLE. We defined depressive symptoms as a negative binomial distributed outcome and tested a direct path from early NLE to depressive symptoms and indirect paths from early NLE to depressive symptoms via the intercept and slope of OFC thickness. We included the control variables sex, site, and emotional symptoms at the first time point. Since the residual variance between persons in the slope parameter was close to zero, the model estimation did not terminate normally. Therefore, we fixed the slope residual variance to zero.

All models were conducted using Mplus version 8.7 with full‐information maximum likelihood estimation (FIML) to account for missing data (Muthén & Muthén, 2017). In Figure S4, we present the patterns of missing data for OFC thickness in the final sample.

## RESULTS

### Descriptive results of negative life events, depressive symptoms and emotional symptoms

The majority of participants reported at least one early NLE before the first time point (*n* = 319; 99%) with a median score of 6 (*MAD* = 2.97; range: 0–24). Most participants (*n* = 243; 76%) reported subclinical depressive symptomatology at the fourth time point (CES‐D < 16; *Mdn* = 9; *MAD* = 7.41). Emotional symptoms as a proxy for depressive symptoms at the first time point showed low scores (*Mdn* = 2; *MAD* = 2.97; range: 0–9). Details on descriptive statistics and distributions for these measures are provided in Table S3 and Figure S2.

### Orbitofrontal cortical thickness

**FIGURE 1:**
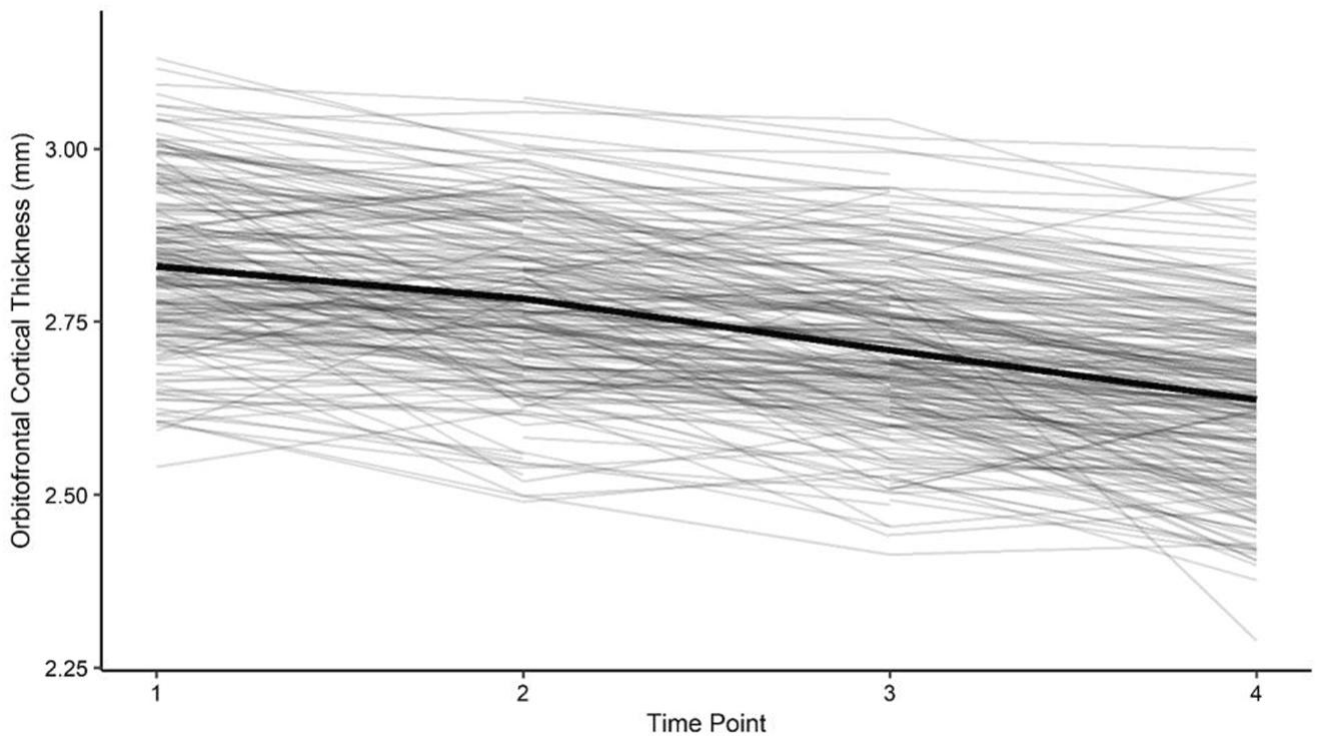
Individual and Mean Trajectories of Cortical Thickness in the Orbitofrontal Cortex. Individual data points are shown connected for each participant, *N* = 321. The thicker black line connects the mean cortical thicknesses from each time point. Participants with single data points (*n* = 23) are not depicted.

Figure 1 illustrates observed individual and mean trajectories of OFC thickness. Herein, a trend towards a linear decrease in OFC thickness across adolescence can be identified. However, a high level of variance between individuals was found. The distribution of OFC thickness per time point is shown in Figure S3.

### Latent growth curve and multiple‐mediators model

For model fit indices and comparisons of the unconditional LGCM modeling of OFC thinning without any covariates, see Table S6. We identified a linear model with homoscedastic residual structure as the best fit for the longitudinal OFC thinning. The retained linear LGCM adequately fit the data [*χ* ^2^(8) = 11.07, CFI = 0.995, TLI = 0.997, RMSEA = 0.035]. The latent slope mean indicates that on average the OFC thickness decreased across adolescence (*μ* = −0.025, *p* < 0.001).

**FIGURE 2:**
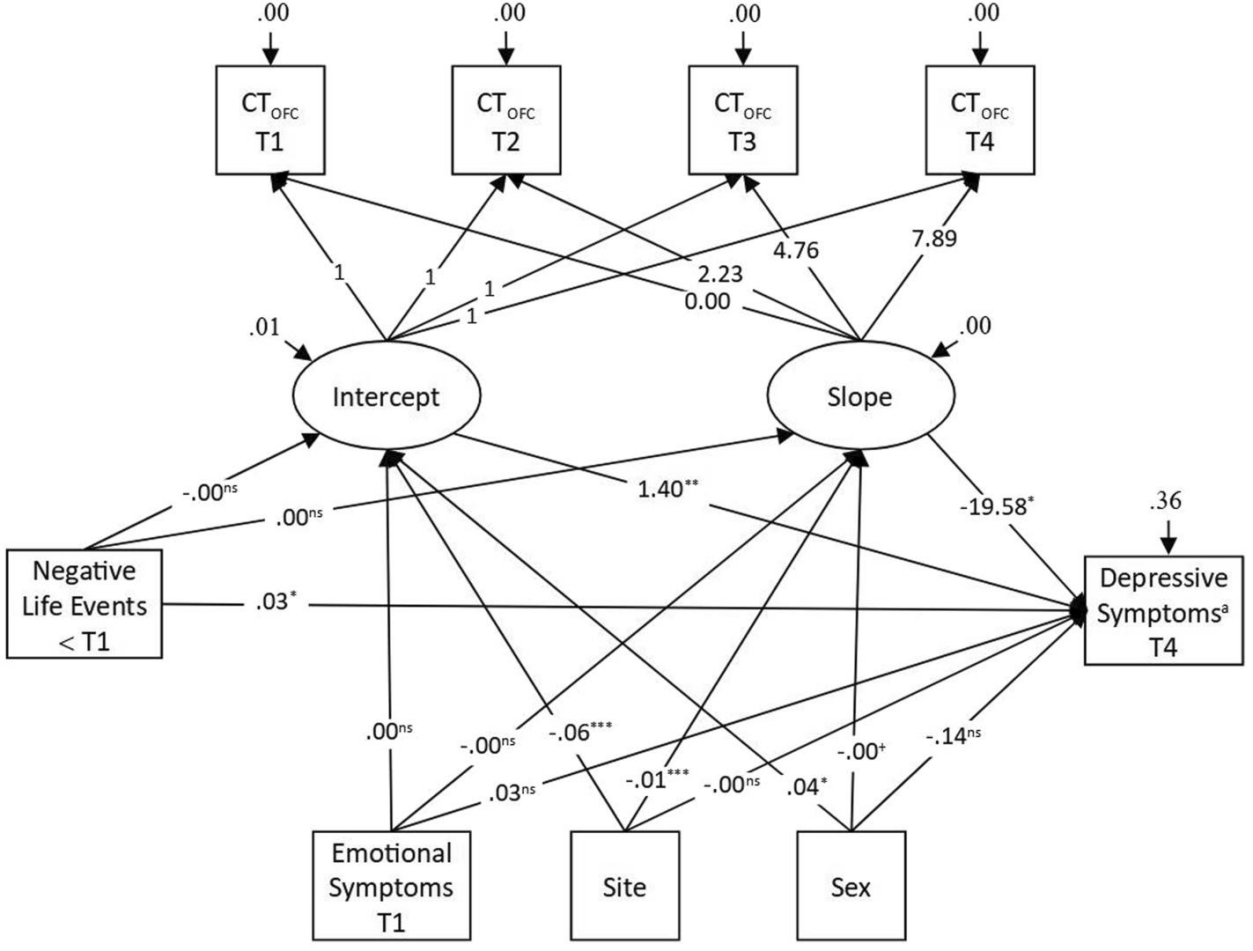
Unstandardized Solution of the Multiple‐Mediators Model. Ovals indicate latent variables, rectangles indicate observed variables, small arcs indicate residuals. Latent variables and covariates were allowed to covary among themselves (not shown). The slope residual variance was set to 0. T1‐T4 = first (baseline) to fourth time point. Depressive symptoms score was measured with the Center for Epidemiologic Studies Depression Scale (CES‐D; Radloff, 1977). Negative life events score was assessed with the Life Events Questionnaire (LEQ; Newcomb et al., 1981). Latent intercept and slope variables represent the cortical thickness in the orbitofrontal cortex at the first time point and its change across adolescence. *p < 0.05; **p < 0.01; ***p < 0.001; ns = non‐significant. ^a^For effects on depressive symptoms negative binomial coefficients are shown.

For the multiple‐mediators model, we included early NLE and control variables (sex, site, emotional symptoms at the first time point) as predictors of the latent intercept and latent slope and regressed depressive symptoms on these latent growth factors and their predictors (see Figure 2 and Table S7). Because the regression coefficients scale differently due to the varying distribution of mediators and depressive symptoms, we present them unstandardized. For correlations across all measures, see Table S5 and for a more detailed evaluation of model fit (sample and estimated means and proportions), see Figure S5.

**FIGURE 3:**
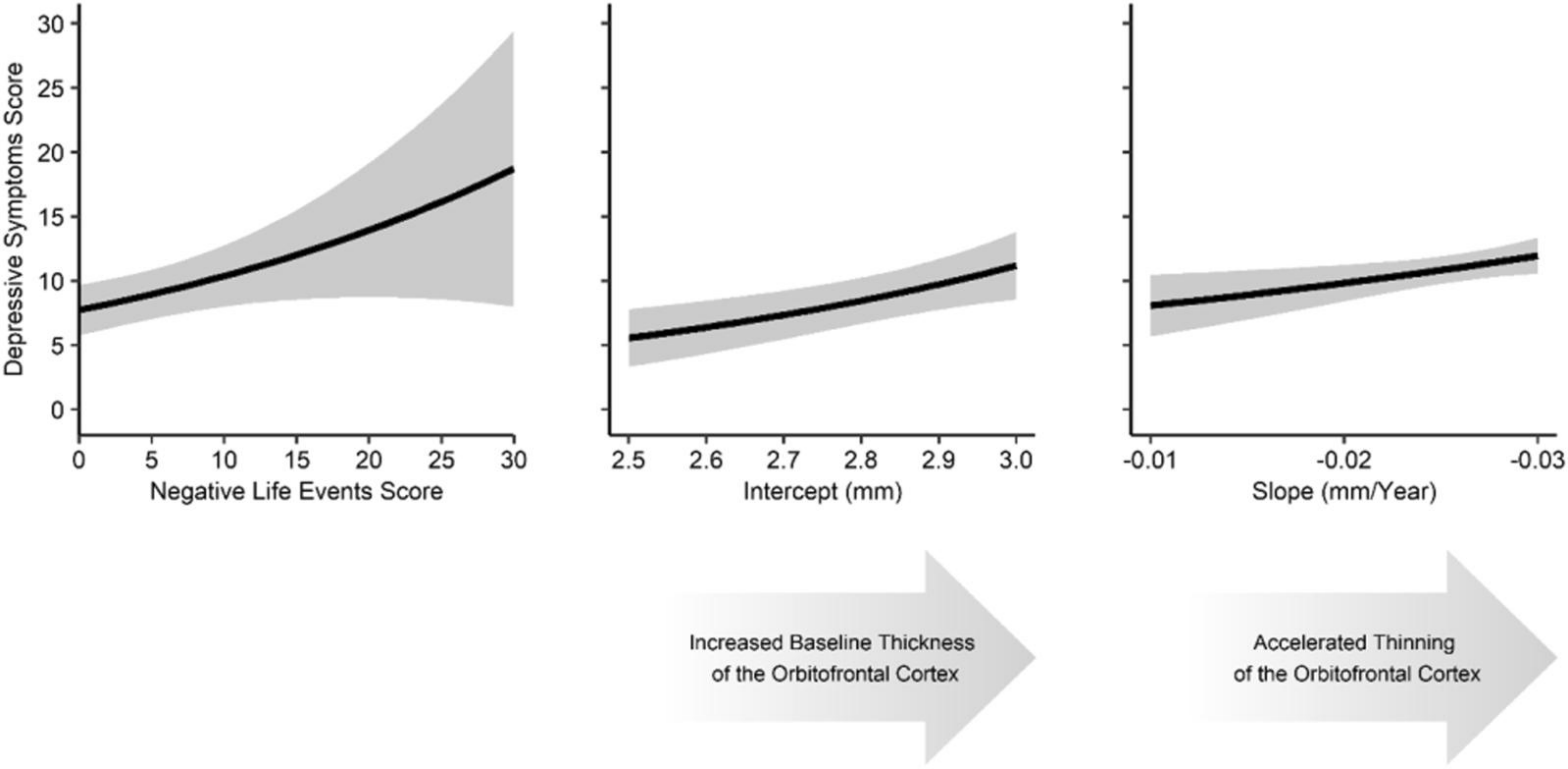
Effects of Negative Life Events and the Development of Cortical Thickness in the Orbitofrontal Cortex Across Adolescence on Depressive Symptoms in Young adulthood. Effects are probed based on the results of the multiple‐mediators model. All covariates except the one plotted are held constant at their mean or median. Latent intercept and slope variables represent the cortical thickness in the orbitofrontal cortex at the first time point and its change across adolescence.

Regarding the effects of interest, participants with a higher burden of early NLE before the first time point tended to have more depressive symptoms at the fourth time point (*B* = 0.03, *IRR* = 1.03, *p* = 0.01; see Figure 3). We found no significant effects of early NLE on OFC thickness at the first time point or on its change across adolescence (see Figure S6). Accordingly, our mediation model (see Table 2) revealed significant total and direct pathways from early NLE to depressive symptoms, but non‐significant indirect pathways via intercept and slope of OFC thickness (see Table 2). Moreover, we found that participants with a greater baseline OFC thickness (*B* = 1.4, *IRR* = 4.052, *p* = 0.004) and an accelerated OFC thinning (*B* = −19.58, *IRR* = 3.13e −9, *p* = 0.026) reported more depressive symptoms (see Figure 3).

**TABLE 2:** Direct and Indirect Effects of Negative Life Events on Depressive Symptoms.

Effect	Estimate	SE	Est./SE	*p*
Total	0.023	0.011	2.122	0.034
Total indirect	−0.006	0.006	−1.047	0.295
Specific indirect				
NLE ‐ intercept ‐ depressive symptoms	−0.003	0.002	−1.302	0.193
NLE ‐ slope ‐ depressive symptoms	−0.003	0.005	−0.622	0.534
Direct				
NLE ‐ depressive symptoms	0.030	0.011	2.587	0.01

*Note*: Latent variables (intercept and slope) represent the development of cortical thickness in the orbitofrontal cortex across adolescence. We assessed depressive symptoms with the Center for Epidemiological Studies Depression Scale (CES‐D; Radloff, 1977) and negative life events (NLE) with the Life Events Questionnaire (LEQ; Newcomb et al., 1981).

Regarding covariates, significant effects of sex and site on OFC thickness were found. Male compared to female participants showed a thicker OFC at the first time point (*B* = 0.039, *p* = 0.002) while the thinning across adolescence did not differ between sexes. Participants from Site 2 compared to those from Site 1showed a thinner OFC at the first time point (*B* = −0.063, *p* < 0.001) and accelerated thinning (*B* = −0.012, *p* < 0.001). There were no effects of sex and site on depressive symptoms and no effects of initial emotional symptoms on OFC thickness nor depressive symptoms in young adulthood (see e.g. Figure S7).

## DISCUSSION

This study is the first to examine the long‐term effect of early NLE on OFC thinning across adolescence and, in turn, on depressive symptoms in young adulthood in a complete longitudinal design with four structural MRI measurements. We tested a LGCM with the development of OFC thickness as a potential mediator between early NLE and depressive symptoms. Our results provide evidence for the impact of early NLE and alterations in OFC thinning during adolescence on the development of depressive symptoms thereafter.

Our MRI assessment of four waves in a complete longitudinal design allowed to precisely model OFC thickness trajectories across adolescence. As a result, we identified linear thinning of the OFC from age 14 into young adulthood. However, contrary to our first hypothesis, we did not find that the experience of early NLE significantly influenced OFC thinning into young adulthood. Still, in line with our second hypothesis, accelerated linear OFC thinning across adolescence led to increased depressive symptoms in young adulthood. We also found that a thicker OFC at age 14 had the same effect. Third, on a psychopathological level, we observed more depressive symptoms in young adulthood in individuals who experienced heightened early NLE. Lastly, we could not confirm our hypothesis regarding OFC thinning mediating the effect of early NLE on depressive symptoms in young adulthood.

### No effect of early negative life events on orbitofrontal cortex thinning

Contrary to our hypothesis, early NLE did not affect OFC thinning across adolescence. A recent study found an association of early NLE, which were present at least for 3 months during infancy, with reduced OFC thickness in 25‐year olds (Monninger et al., 2020). However, unlike in our study, events during infancy were investigated via parent report, which do not necessarily reflect individual perception of these events. Further, Monninger et al. (2020) only performed one measurement in 190 25‐year‐olds, and could therefore not examine the influences of early NLE influences on longitudinal OFC development. This and the fact that depressive symptoms were measured a few years prior to OFC thickness whereas we assessed them after OFC development makes it difficult to compare results of Monninger et al. (2020) with ours.

Putting our results within the framework of the Stress Acceleration Hypothesis, which presents a neurobiological framework for increased vulnerability to psychopathology being the long‐term consequence of brain adaptions (Callaghan & Tottenham, 2016), remains complicated. Childhood adversity (more severe events than the ones we measured) seems to disturb further development and might have thus led to greater alterations in (prefrontal) cortical development (McLaughlin et al., 2019; Schmaal et al., 2020). Speculatively, similar effects might have taken place after the experience of early NLE, which future studies may detect in a bigger sample with higher statistical power. Further, because we only examined the OFC as a region of interest we were unable to uncover possible impacts of early NLE on other brain regions, notably the amygdala and hippocampus. Alterations in those regions have been repeatedly found to be associated with adversity in childhood (McLaughlin et al., 2019; Pollok et al., 2022). However, as maturation of these subcortical regions is mostly complete until adolescence (Dennison et al., 2013; Wierenga et al., 2018) we did not include them in this study. Based on results from recent meta‐analyses and reviews, other cortical regions of interest like the inferior frontal gyrus, insula, anterior cingulate cortex, and dorsolateral prefrontal cortex seem to be promising as well when further investigating the effect on early NLE on brain development (McLaughlin et al., 2019; Paquola et al., 2016; Pollok et al., 2022).

### Effects of early negative life events on depressive symptoms

Our results are partly in line with Monninger et al. (2020) who also investigated the interplay between the three factors NLE, OFC thickness and depressive symptoms and showed a significant relationship between early NLE in infancy (e.g. parental disharmony or illness of a relative) and depressive symptoms in adulthood. We showed that early NLE not only affect the development of depressive symptoms when experienced during infancy (up to age 4; see Monninger et al., 2020) but also in later childhood and adolescence. By using the LEQ we also took challenging events typically experienced in early to mid‐ adolescence, like problems with family, friends and sexuality, or social and academic problems into account. Together with the finding of Monninger et al. (2020) our results extend previous research by showing that early NLE might predict depressive symptoms in the long term, similarly as severe NLE like childhood adversity (e.g. Li et al., 2016; McLaughlin, 2016).

### Effects of orbitofrontal cortex thickness on depressive symptoms

Using a complete longitudinal design, we showed that accelerated OFC thinning across adolescence predicted heightened depressive symptoms in the long term, that is, in young adulthood. This critically extends previous findings of accelerated frontal cortical thinning across adolescence predicting depression 4 years later (Bos et al., 2018). Our results contradict findings of Ducharme et al. (2014) of slower ventromedial prefrontal cortex thinning in children and adolescents with higher anxious/depressed symptoms. However, Ducharme et al. (2014) used the Child Behavior Checklist, so their score contains slightly different symptoms than Bos et al. (2018) using the Beck's Depression Inventory and our study using the CES‐D, limiting comparability of results. Further, both studies used an accelerated longitudinal design including a broad age span from around five to 22 years of age, where however each participant is only followed over a shorter time span relative to the entire age span of interest, leading to missing data. With our four time points spanning the whole age span of interest we were able to precisely characterize OFC thinning during adolescence and investigate effects on depressive symptoms.

### No mediation of early NLE effects on depressive symptoms via orbitofrontal cortex thinning

Our model suggests that the influence of early NLE on depressive symptoms in adulthood is not mediated by the development of the OFC. Still, we revealed that both early NLE and alterations in OFC thinning across adolescence predicted depressive symptoms in young adulthood directly and independently. We thus conclude that these two effects act through distinct pathways. Therefore, our study cannot resolve the underlying factors which led to accelerated thinning in the OFC and thus to depressive symptoms. To discuss possible candidates, a recent meta‐analysis identified low birth weight, parental adversities and environmental adversities like low socioeconomic status (SES) and air pollution to alter frontal lobe volumes in adolescents and young adults (Pollok et al., 2022). Regarding longitudinal influences of SES, Piccolo et al. found curvilinear rather than linear decrease in cortical thickness from childhood to young adulthood in lower levels of SES (2016). As in our sample, the vast majority of participants grew up high SES household, this factor is unlikely to have led to differences in cortical development. Our results in connection with other recent findings (Pollok et al., 2022), Piccolo et al. (2016) seem to indicate that the effects of early NLE work temporal, qualitative, quantitative (dose‐dependent) and in interaction with individual dispositions (including resilience from compensation capacity, e.g. cognitive and emotional ability, genetic disposition, stress coping skills) and may have different and sometimes even opposite effects on OFC thickness development and other outcomes.

### Limitations

While this study had its strengths like a four‐wave complete longitudinal design with a high density of MRI data points in the investigated age range, some limitations need to be acknowledged. First, early NLE scores were in the lower range overall (median of 6 on a scale of 0 to 78). This may have contributed to the lack of effect on OFC thinning. Second, several characteristics of early NLE such as timing, frequency, duration, and predictability could not be accounted for. As brain regions have sensitive periods regarding the impact of stress (McLaughlin et al., 2019), important dynamic effects could not be considered in this study. Third, we did not differentiate between dimensions of early NLE. Different stressors may have different effects on brain structure (McLaughlin et al., 2019) and psychological development. The questionnaire used in this study covered a wide range of events, presumably of varying severity (e.g. “Death in family”, “Parents divorced”, as well as “Got poor grades at school” or “Face broke out with pimples”). As a result, the scale may not have been optimally sensitive to the outcome measures of OFC thinning and depressive symptoms. However, we did take into account subjective distress of the events, as experienced events were weighted higher if the subsequent feeling was reported as “very unhappy” compared to “unhappy”. Nevertheless, a higher total score of early NLE could also represent a higher individual sensitivity to stressful experiences. This, in turn could be a common factor for a higher total score of early NLE and an increase in depressive symptoms (perhaps introducing a response bias in the questionnaire data). This is consistent with the diathesis‐stress models of depression, which suggest that vulnerabilities in genetic, physiological, and cognitive or affective systems predispose individuals to stress sensitivity, thereby increasing the likelihood of depression onset when experiencing stress (Monroe & Cummins, 2015). Regarding the assessment of early NLE, it should be noted that the occurrence and valence of early NLE were self‐reported retrospectively at age 14 and thus may be subject to recall bias. Furthermore, several influential variables (e.g. temperament; Gonda et al., 2020) and other risk factors could have been a potential latent factor for the relationship between early NLE and depressive symptoms later in life.

Finally, although we controlled for site in the analyses, we acknowledge that multisite longitudinal studies add noise to the data, which must be considered a limitation. However, because the same scanner manufacturer was used at all sites in this study, we consider this to be within acceptable limits.

## CONCLUSION

Taken together, current findings extend previous studies by showing that early NLE can predict depressive symptoms in the long term. Moreover, using a complete longitudinal design with four waves, results indicate that accelerated OFC thinning may precede depressive symptoms giving new insight into the neurodevelopmental factors associated with the development of depression. Our results suggest that NLE assessment in childhood and adolescence in addition to acute NLE may be warranted in clinical psychology and psychotherapy to identify individuals at risk for depression. Additionally, accelerated thinning of prefrontal cortical areas may be an additional risk factor for the development of depressive symptoms, which should receive further attention in efforts to prevent psychological disorders in young adults. Finally, future studies could follow up on the complex interplay of factors which may promote resilient functioning, as those mitigate the adverse effects of NLE. These comprise for example, community support, cultural resources and peer/family support (see Ioannidis et al., 2020), positive coping strategies and optimism (see Holz et al., 2020).

## AUTHOR CONTRIBUTIONS

**Lea L. Backhausen**: Formal analysis, Methodology, Visualization, Writing – original draft. **Jonas Granzow**: Formal analysis, Methodology, Visualization, Writing – review & editing. **Juliane H. Fröhner**: Data curation, Project administration, Writing – review & editing. **Eric Artiges**: Validation, Writing – review & editing. **Marie‐Laure Paillère Martinot**: Funding acquisition, Validation, Writing – review & editing. **Hervé Lemaitre**: Data curation, Writing – review & editing. **Fabio Sticca**: Methodology, Writing – review & editing. **Jean‐Luc Martinot**: Conceptualization, Funding acquisition, Validation, Writing – review & editing. **Michael Smolka**: Conceptualization, Funding acquisition, Writing – review & editing. **Nora Vetter**: Conceptualization, Funding acquisition, Methodology, Project administration, Supervision, Writing – original draft.

## CONFLICT OF INTEREST STATEMENT

Dr. Banaschewski served in an advisory or consultancy role for Lundbeck, Medice, Neurim Pharmaceuticals, Oberberg GmbH, Shire. He received conference support or speaker's fee by Lilly, Medice, Novartis and Shire. He has been involved in clinical trials conducted by Shire & Viforpharma. He received royalties from Hogrefe, Kohlhammer, CIP Medien, Oxford University Press. The present work is unrelated to the above grants and relationships. Dr. Poustka served in an advisory or consultancy role for Roche and Viforpharm and received speaker's fee by Shire. She received royalties from Hogrefe, Kohlhammer and Schattauer. The present work is unrelated to the above grants and relationships. The other authors report no biomedical financial interests or potential conflicts of interest.

### OPEN RESEARCH BADGES

This article has earned Open Data and Open Materials badges. Data and materials are available at https://osf.io/5tfh4/ on the Open Research Disclosure Form.

## ETHICAL CONSIDERATIONS

The local ethics committee (TUD Dresden University of Technology and CPP IDF VII ‐Biomedical, CCPPRB Le Kremlin‐Bicetre) approved the study and it was performed in accordance with the Declaration of Helsinki. Participants and, in case of participants younger than 18 years, legal guardians gave written informed consent.

## Supporting information

Supporting Information S1

## Data Availability

Access to the IMAGEN dataset (raw data) is available with an accepted proposal from the IMAGEN executive committee (https://imagen‐project.org/the‐imagen‐dataset/). All preprocessed data and code is posted to Open Science Framework and can be assessed at https://osf.io/5tfh4/.
