# Correction to “Interplay of early negative life events, development of orbitofrontal cortical thickness and depression in young adulthood”

**DOI:** 10.1002/jcv2.12237

**Published:** 2024-04-15

Backhausen, L. L., Granzow, J., Fröhner, J. H., Artiges, E., Paillère‐Martinot, M.‐L., Lemaître, H., Sticca, F., Banaschewski, T., Desrivières, S., Grigis, A., Heinz, A., Brühl, R., Papadopoulos‐Orfanos, D., Poustka, L., Hohmann, S., Robinson, L., Walter, H., Winterer, J., Schumann, G., Martinot, J.‐L., Smolka, M. N., Vetter, N. C., the IMAGEN Consortium. (2024). Interplay of early negative life events, development of orbitofrontal cortical thickness and depression in young adulthood. JCPP Advances, 4(1), e12210. https://doi.org/10.1002/jcv2.12210.

The statement of equal contributions should have been: Lea L. Backhausen and Jonas Granzow (first authors) and Jean‐Luc Martinot and Michael N. Smolka have contributed equally.

We apologize for the error.

