# Supplementary material for: Interplay of early negative life events, development of orbitofrontal cortical thickness and depression in young adulthood
Source: JCPP Adv. 2023 Dec 6;4(1):e12210. doi: 10.1002/jcv2.12210 (PMC10933677; doi:10.1002/jcv2.12210)
Supplement: Supplementary file 1 — Supporting Information S1 [file JCV2-4-e12210-s001.docx]

Supplement 1

# Supplementary Tables

| **Table S1.** Quality Control Flow and Details on the Inclusion and Exclusion of Participants | | | | | | | | | | | |
| --- | --- | --- | --- | --- | --- | --- | --- | --- | --- | --- | --- |
|  | | | | | | | | | | | |
|  | **Site 1** | | | | |  | **Site 2** | | | | |
|  | **Subjects^a^** | **T1** | **T2** | **T3** | **T4** |  | **Subjects^a^** | **T1** | **T2** | **T3** | **T4** |
| Total number of participants, *n* | 260 |  |  |  |  |  | 274 |  |  |  |  |
|  | *Pre-processing* | | | | | | | | | | |
| MPRAGE scans available, *n* | 256 | 235 | 218 | 180 | 138 |  | 258 | 257 | 138 | 205 | 123 |
| Pre-processing QC fail, *n* | 8 | 33 | 11 | 7 | 8 |  | 3 | 11 | 8 | 4 | 1 |
| Available for FS processing, *n* | 248 | 202 | 207 | 173 | 130 |  | 255 | 246 | 130 | 201 | 122 |
| Successfully processed, n | 248 | 202 | 207 | 173 | 130 |  | 255 | 246 | 130 | 201 | 122 |
|  | *Post-processing^b^* | | | | | | | | | | |
| Post-processing QC fail, *n* | 4 | 35 | 18 | 10 | 12 |  | 2 | 3 | 3 | 9 | 4 |
|  | *Exclusions^c^* | | | | | | | | | | |
| No LEQ available, *n* | 1 | 1 | 1 | 1 | 1 |  | 1 | 1 | 1 | 0 | 0 |
| No CES-D available, *n* | 84 | 58 | 55 | 40 | 4 |  | 90 | 88 | 30 | 49 | 1 |
|  | *Final data set* | | | | | | | | | | |
| Number of subjects/  scans per time point | 159 | 108 | 133 | 122 | 113 |  | 162 | 154 | 96 | 143 | 117 |
|  | | | | | | | | | | | |
| ***Note.*** One of three trained operators visual quality controlled each scan. For scans of questionable quality further visual quality control was applied after FreeSurfer processing. T1-T4 = first (baseline) to fourth time point; MPRAGE = magnetization prepared rapid acquisition gradient-echo sequence; QC = quality control; FS = FreeSurfer.  ^a^ Total number of subjects for whom a (high-quality) scan for at least one time point was available.  ^b^ Single scans were excluded post-processing when the cortex was parcellated incorrectly by FreeSurfer.  ^c^ Complete data sets from participants were excluded if they did not complete the Life Events Questionnaire (LEQ; Newcomb et al., 1981) at the first time point and Center for Epidemiologic Studies Depression Scale (CES-D; Radloff, 1977) at the fourth time point. | | | | | | | | | | | |

| **Table S2.** Sample Characteristics Including Demographics of Typically Developing Adolescents (Separated by Site) | | | | | | | | | |
| --- | --- | --- | --- | --- | --- | --- | --- | --- | --- |
|  | | | | | | | | | |
| **Variable** | **Site 1** | | |  | **Site 2** | | | **Test Statistics** | |
|  | **All** | **Female** | **Male** |  | **All** | **Female** | **Male** |  |  |
| Number of participants, *n* | 159 | 83 | 76 |  | 162 | 92 | 70 |  |  |
| *Age (years)^a^* | | | | | | | | | |
|  |  |  |  |  |  |  |  | ***t*** | ***p*** |
| T1, *M* (*SD*) | 14.5 (0.3) | 14.6 (0.3) | 14.4 (0.4) |  | 14.4 (0.5) | 14.4 (0.5) | 14.4 (0.5) | 2.51 | .013 |
| T2, *M* (*SD*) | 16.6 (0.4) | 16.6 (0.4) | 16.5 (0.4) |  | 16.8 (0.6) | 16.9 (0.6) | 16.7 (0.6) | -3.23 | .002 |
| T3, *M* (*SD*) | 18.7 (0.6) | 18.7 (0.5) | 18.6 (0.6) |  | 19.7 (0.7) | 19.7 (0.7) | 19.6 (0.7) | -12.93 | < .001 |
| T4, *M* (*SD*) | 22.1 (0.7) | 22.1 (0.6) | 22.0 (0.8) |  | 22.6 (0.5) | 22.6 (0.5) | 22.6 (0.5) | -6.76 | < .001 |
| *Demographics* | | | | | | | | | |
|  |  |  |  |  |  |  |  | ***χ^2^*** | ***p*** |
| Sex (female), *n* (*%*) | 83 (52.2%) |  |  |  | 92 (56.8%) |  |  | 0.51 | .476 |
| Handedness (right-handed), *n* (*%*)^b^ | 142 (89.3%) | 75 (90.4%) | 67 (88.2%) |  | 141 (88.1%) | 83 (90.2%) | 58 (85.3%) | 0.02 | .875 |
| Non-white ethnicity, *n* (*%*) | 3 (1.9%) | 2 (2.4%) | 1 (1.3%) |  | 14 (8.7%) | 9 (9.9%) | 5 (7.1%) | 6.08 | .014 |
|  |  |  |  |  |  |  |  | ***U*** | ***p*** |
| Height in cm, *M* (*SD*) | 169.8 (7.1) | 167.3 (6.3) | 172.5 (6.9) |  | 165.2 (7.6) | 162.8 (5.5) | 168.4 (8.7) | 17036.0 | < .001 |
| Weight in kg, *M* (*SD*) | 59.7 (11.7) | 57.3 (10.0) | 62.4 (12.8) |  | 54.7 (9.0) | 53.3 (7.5) | 56.5 (10.5) | 15860.5 | < .001 |
| BMI, *M* (*SD*) | 20.6 (3.2) | 20.4 (3.0) | 20.8 (3.5) |  | 20.0 (2.6) | 20.1 (2.5) | 19.8 (2.8) | 13868.5 | .109 |
| IQ^c^, *M* (*SD*) | 115.2 (10.4) | 115.3 (9.4) | 115.0 (11.4) |  | 110.5 (8.8) | 109.8 (7.9) | 111.4 (9.9) | 15564.0 | < 0.001 |
| Pubertal status^d^, *Md* (*MAD*) | 4 (0.0) | 4.0 (0.0) | 3 (0.0) |  | 4 (0.0) | 4 (0.0) | 3 (1.5) | 13569 | .287 |
| AUDIT, *Md* (*MAD*) | 1 (1.5) | 1 (1.5) | 0 (0.0) |  | 0 (0.0) | 0 (0.0) | 0 (0.0) | 12988.5 | .723 |
| SDQ emotional symptoms, *Md* (*MAD*) | 3 (3.0) | 4 (1.5) | 2 (1.5) |  | 2 (3.0) | 3 (3.0) | 1 (1.5) | 14089.5 | .141 |
| LEQ, *Md* (*MAD*) | 7 (4.4) | 8 (4.4) | 6 (3.0) |  | 5 (3.0) | 6 (3.0) | 5 (3.0) | 16397.5 | < .001 |
| CES-D (T4), *Md* (*MAD*) | 8 (5.9) | 9 (7.4) | 8 (4.4) |  | 9 (7.4) | 10 (8.2) | 8 (5.9) | 12174 | .396 |
|  | | | | | | | | | |

| **Table S2** (continued)  ***Note.*** Data is reported for all participants in the final data set independent of a high-quality scan at the first time point. Unless otherwise stated, statistics are given for the first time point. Please note that sociodemographic information was missing for few participants (handedness: *n* = 2; ethnicity: *n* = 1; weight, height and BMI: *n* = 4, IQ: *n* = 10; pubertal status: *n* = 1; alcohol use: *n* = 2). The percentages indicated refer to the available information. T1-T4 = first (baseline) to fourth time point; BMI = body mass index; AUDIT = Alcohol Use Disorders Identification Test (Saunders et al., 1993); SDQ = Strengths and Difficulties Questionnaire (Goodman, 1997); LEQ = Life Event Questionnaire (Newcomb et al., 1981); CES-D = Center for Epidemiologic Studies Depression Scale (Radloff, 1977).  ^a^ For information on age, only participants with a scan included at the respective time point were considered.  ^b^ Remaining participants reported left-handedness except for one participant per site who reported ambidextrous-handedness. Information on handedness was missing for two participants from site 2.  ^c^ General cognitive ability estimated with the subtests Similarities, Block Design, Vocabulary, and Matrices from the Wechsler Intelligence Scale for Children Fourth Edition (WISC®-IV; Wechsler, D., 2003).  ^d^ Pubertal status ranged from 1 for ‘prepubertal’ to 5 for ‘postpubertal status’, measured with the Pubertal Development Scale (PDS; Petersen et al., 1988). The value 4 corresponding to the median represents ‘advanced pubertal’. |
| --- |

| **Table S3.** Descriptive Statistics for Negative Life Events, Emotional Symptoms and Depressive Symptoms | | | | | | | | | | |
| --- | --- | --- | --- | --- | --- | --- | --- | --- | --- | --- |
|  |  |  |  |  |  |  |  |  |  |  |
| **Variable** | ***N*** | ***M*** | ***SD*** | ***Mdn*** | ***MAD*** | **Min** | **Max** | **Skew** | **Kurtosis** | ***SE*** |
|  | *Negative Life Events (LEQ)* | | | | | | | | | |
| Total | 321 | 6.59 | 3.97 | 6 | 2.97 | 0 | 24 | 0.96 | 1.49 | 0.22 |
| Site 1 | 159 | 7.65 | 4.45 | 7 | 4.45 | 0 | 24 | 0.8 | 0.83 | 0.35 |
| Site 2 | 162 | 5.55 | 3.12 | 5 | 2.97 | 0 | 16 | 0.62 | 0.47 | 0.25 |
|  | *Emotional Symptoms (SDQ emotional symptoms score)* | | | | | | | | | |
| Total | 321 | 2.62 | 2.21 | 2 | 2.97 | 0 | 9 | 0.7 | -0.13 | 0.12 |
| Site 1 | 159 | 2.76 | 2.14 | 3 | 2.97 | 0 | 9 | 0.65 | 0.08 | 0.17 |
| Site 2 | 162 | 2.48 | 2.27 | 2 | 2.97 | 0 | 9 | 0.77 | -0.29 | 0.18 |
|  | *Depressive Symptoms (CES-D)* | | | | | | | | | |
| Total | 321 | 11.25 | 8.59 | 9 | 7.41 | 0 | 49 | 1.51 | 2.63 | 0.48 |
| Site 1 | 159 | 10.67 | 7.96 | 8 | 5.93 | 0 | 43 | 1.61 | 2.88 | 0.63 |
| Site 2 | 162 | 11.83 | 9.15 | 9 | 7.41 | 0 | 49 | 1.39 | 2.22 | 0.72 |
|  | | | | | | | | | | |
| ***Note****.* Negative Life Events and emotional symptoms were measured at the first time point, depressive symptoms at the fourth time point. MAD = median absolute deviation; Min = minimum; Max = maximum; Skew = skewness; LEQ = Life Events Questionnaire (Newcomb et al., 1981); SDQ = Strengths and Difficulties Questionnaire (Goodman, 1997); CES-D = Center for Epidemiologic Studies Depression Scale (Radloff, 1977) | | | | | | | | | | |

| **Table S4.** Items of the Life Events Questionnaire and Their Frequencies and Percentages in the Total Sample | | | | |
| --- | --- | --- | --- | --- |
|  | | | | |
| Item no. | Item | “Unhappy” or “Very unhappy” | “Unhappy“ | “Very unhappy” |
| 1 | Parents divorced | 40 (12.46%) | 26 (8.1%) | 14 (4.36%) |
| 2 | Family accident or illness | 180 (56.07%) | 98 (30.53%) | 82 (25.55%) |
| 3 | Found a new group of friends | 5 (1.56%) | 4 (1.25%) | 1 (0.31%) |
| 4 | Got into trouble with the law | 12 (3.74%) | 8 (2.49%) | 4 (1.25%) |
| 5 | Stole something valuable | 6 (1.87%) | 2 (0.62%) | 4 (1.25%) |
| 6 | Given medication by physisian | 15 (4.67%) | 14 (4.36%) | 1 (0.31%) |
| 7 | Fell in love | 7 (2.18%) | 3 (0.93%) | 4 (1.25%) |
| 8 | Death in family | 187 (58.26%) | 33 (10.28%) | 154 (47.98%) |
| 9 | Face broke out with pimples | 88 (27.41%) | 69 (21.5%) | 19 (5.92%) |
| 10 | Brother or sister moved | 30 (9.35%) | 24 (7.48%) | 6 (1.87%) |
| 11 | Started seeing a therapist | 2 (0.62%) | 2 (0.62%) | 0 (0%) |
| 12 | Parents changed jobs | 4 (1.25%) | 4 (1.25%) | 0 (0%) |
| 13 | Began a time-consuming hobby | 10 (3.12%) | 7 (2.18%) | 3 (0.93%) |
| 14 | Got or made pregnant | 1 (0.31%) | 1 (0.31%) | 0 (0%) |
| 15 | Decided about college/university | 2 (0.62%) | 2 (0.62%) | 0 (0%) |
| 16 | Thought about suicide | 42 (13.08%) | 17 (5.3%) | 25 (7.79%) |
| 17 | Changed schools | 72 (22.43%) | 55 (17.13%) | 17 (5.3%) |
| 18 | Joined a club or group | 5 (1.56%) | 4 (1.25%) | 1 (0.31%) |
| 19 | Got into trouble at school | 112 (34.89%) | 91 (28.35%) | 21 (6.54%) |
| 20 | Got or gave sexually transmitted disease | 0 (0%) | 0 (0%) | 0 (0%) |
| 21 | Met a teacher I liked | 3 (0.93%) | 3 (0.93%) | 0 (0%) |
| 22 | Family had money problems | 59 (18.38%) | 49 (15.26%) | 10 (3.12%) |
| 23 | Got own TV or computer | 3 (0.93%) | 0 (0%) | 3 (0.93%) |
| 24 | Parents argued or fought | 130 (40.5%) | 75 (23.36%) | 55 (17.13%) |
| 25 | Ran away from home | 10 (3.12%) | 3 (0.93%) | 7 (2.18%) |
| 26 | Started going out with a girlfriend/boyfriend | 0 (0%) | 0 (0%) | 0 (0%) |
| 27 | Got poor grades at school | 206 (64.17%) | 167 (52.02%) | 39 (12.15%) |
| 28 | Went on holiday without parents | 3 (0.93%) | 3 (0.93%) | 0 (0%) |
| 29 | Started driving a motor vehicle | 0 (0%) | 0 (0%) | 0 (0%) |
| 30 | Broke up with boy/girl friend | 127 (39.56%) | 88 (27.41%) | 39 (12.15%) |
| 31 | Family moved | 67 (20.87%) | 53 (16.51%) | 14 (4.36%) |
| 32 | Started making own money | 0 (0%) | 0 (0%) | 0 (0%) |
| 33 | Found religion | 0 (0%) | 0 (0%) | 0 (0%) |
| 34 | Parents remarried | 0 (0%) | 0 (0%) | 0 (0%) |
| 35 | Had a gay experience | 0 (0%) | 0 (0%) | 0 (0%) |
| 36 | Gained a lot of weight | 57 (17.76%) | 31 (9.66%) | 26 (8.1%) |
| 37 | Serious accident or illness | 43 (13.4%) | 28 (8.72%) | 15 (4.67%) |
| 38 | Lost virginity | 1 (0.31%) | 1 (0.31%) | 0 (0%) |
| 39 | Parents abused alcohol | 18 (5.61%) | 14 (4.36%) | 4 (1.25%) |
|  |  |  |  |  |
| ***Note.*** Negative life events before the first time point were assessed with the Life Events Questionnaire (LEQ; Newcomb et al., 1981), N = 321. For each item the total number and percentage of participants who stated that this event has ever occurred in their lives and rated the feeling in response to that event as “unhappy” and/or “very unhappy” are reported. | | | | |

| **Table S5.** Complete Correlation Matrix for Variables of Interest Included in the Final Model | | | | | | | | |
| --- | --- | --- | --- | --- | --- | --- | --- | --- |
|  | | | | | | | | |
|  | **1** | **2** | **3** | **4** | **5** | **6** | **7** | **8** |
| **1. Sex** | 1 |  |  |  |  |  |  |  |
| **2. Site** | -.046 | 1 |  |  |  |  |  |  |
| **3. Emotional Symptoms** | -.311 | -.063 | 1 |  |  |  |  |  |
| **4. Negative Life Events** | -.137 | -.265 | .211 | 1 |  |  |  |  |
| **5. CT_OFC_ (T1)** | .151 | -.239 | -.024 | -.017 | 1 |  |  |  |
| **6. CT_OFC_ (T2)** | .242 | -.337 | -.109 | -.011 | .746 | 1 |  |  |
| **7. CT_OFC_ (T3)** | .132 | -.563 | -.026 | .090 | .728 | .810 | 1 |  |
| **8. CT_OFC_ (T4)** | .089 | -.567 | -.068 | .088 | .727 | .749 | .822 | 1 |
|  | | | | | | | | |
| ***Note*.** Reported correlations are based on FIML estimation for missing data. Since depressive symptoms followed a negative binomial distribution, correlations of this variable are not shown. Sex: 0 = „female“, 1 = „male“; Site: 0 = Site 1, 1 = Site 2; „Emotional Symptoms“ = SDQ Emotional Symptoms Score (Goodman, 1997); „Negative Life Events“ = Negative Life Events Score measured with the Life Events Questionnaire (LEQ; Newcomb et al., 1981b). CT_OFC_ (T1) - CT_OFC_ (T4) = Orbitofrontal Cortical Thickness at the first, second, third, and fourth time point. | | | | | | | | |

| **Table S6.** Model Fit Indices and Results for Model Comparisons of Unconditional Latent Growth Curve Models of Orbitofrontal Cortical Thickness | | | | | | | | | | | | | |
| --- | --- | --- | --- | --- | --- | --- | --- | --- | --- | --- | --- | --- | --- |
|  | | | | | | | | | | | | | |
| **Model** | **Model No.** | **Residual structure** | **df** | **χ^2^** | **CFI** | **TLI** | **SRMR** | **RMSEA** | **AIC** | **BIC** | **Test** | **L Ratio** | ***p*** |
| null | 1 |  | 8 | 664.64 | 0.024 | 0.268 | 2.112 | 0.506 | -1380.02 | -1357.39 |  |  |  |
| linear | 2 | heteroscedastic | 5 | 6.19 | 0.998 | 0.998 | 0.061 | 0.027 | -2032.47 | -1998.53 | 1 vs 2 | 658.46 | < .001 |
| quadratic | 3 | heteroscedastic | 1 | 0.08 | 1.000 | 1.008 | 0.006 | 0.000 | -2030.58 | -1981.55 | 2 vs 3 | 6.10 | .192 |
| linear | 4 | homoscedastic | 8 | 11.07 | 0.995 | 0.997 | 0.114 | 0.035 | -2033.59 | -2010.96 | 2 vs 4 | 4.89 | .180 |
|  | | | | | | | | | | | | | |
| *Note.* The table shows comparisons of different models that predict orbitofrontal cortical thickness. Factor loadings represent the average interval between time points in years, [0 2.23 4.76 7.89]. Likelihood ratio test was performed for significance testing. CFI = Comparative Fit Index; TLI = Tucker-Lewis Index; SRMR = Standardized Root Mean Square Residual; RMSEA = Root Mean Square Error of Approximation; AIC = Akaike Information Criterion; BIC = Bayesian Information Criterion; L Ratio = Likelihood Ratio. | | | | | | | | | | | | | |

| **Table S7.** Estimated Coefficients of the Multiple-Mediators Model | | | | | | | | | |
| --- | --- | --- | --- | --- | --- | --- | --- | --- | --- |
|  | | | | | | | | | |
| **Variable** | ***Estimate*** | | ***SE*** | | ***Est./SE*** |  | | ***p*** | |
| Intercept ~ |  | |  | |  |  | |  | |
| Sex (male) | 0.039 | | 0.012 | | 3.140 |  | | .002 | |
| Site (Site 2) | -0.063 | | 0.012 | | -5.156 |  | | < .001 | |
| Negative life events, LEQ | -0.002 | | 0.002 | | -1.455 |  | | .146 | |
| Emotional symptoms, SDQ | 0.000 | | 0.003 | | 0.056 |  | | .956 | |
| Slope ~ |  | |  | |  |  | |  | |
| Sex (male) | -0.003 | | 0.002 | | -1.890 |  | | .059 | |
| Site (Site 2) | -0.012 | | 0.002 | | -6.682 |  | | < .001 | |
| Negative life events, LEQ | 0.000 | | 0.000 | | 0.649 |  | | .516 | |
| Emotional symptoms, SDQ | -0.001 | | 0.000 | | -1.603 |  | | .109 | |
|  | ***Estimate*** | | ***SE*** | | ***Est./SE*** | ***IRR*** | | ***p*** | |
| Depressive Symptoms ~ |  | |  | |  |  | |  | |
| Sex (male) | -0.144 | | 0.098 | | -1.468 | 0.866 | | 0.142 | |
| Site (Site 2) | 0.000 | | 0.137 | | 0.000 | 1.000 | | 1.000 | |
| Negative life events, LEQ | 0.030 | | 0.011 | | 2.587 | 1.030 | | .010 | |
| Emotional symptoms, SDQ | 0.030 | | 0.021 | | 1.474 | 1.031 | | .140 | |
| Intercept | 1.399 | | 0.480 | | 2.918 | 4.052 | | .004 | |
| Slope | -19.582 | | 8.809 | | -2.223 | 3.13e- 9 | | .026 | |
|  |  |  | |  | | |  | |  |
| ***Note.*** Estimates are shown unstandardized and are differently scaled: Regressions of intercept and slope on predictors are linear; regressions of depression symptoms on predictors are negative binomial (logarithmic). Intercept/Slope = latent variables representing the development of orbitofrontal cortical thickness across adolescence. Depressive symptoms were assessed at the fourth time point with the Center for Epidemiological Studies Depression Scale (CES-D; Radloff, 1977). LEQ = Life Events Questionnaire (Newcomb et al., 1981); SDQ = Strengths and Difficulties Questionnaire (Goodman, 1997); IRR = incidence rate ratio (e^Est.^). | | | | | | | | | |

| **Table S8.** Description of scanning parameters for each site and wave. | | | | | | | | | | |
| --- | --- | --- | --- | --- | --- | --- | --- | --- | --- | --- |
|  |  |  | |  |  |  |  |  |  |  |
| **Site** | **Wave** | **Location** | **Manu-facturer** | | **Platform** | **Field (T)** | **Head Coil** | **TR (ms)** | **TE (ms)** | **voxel size** |
| 1 | 1 | X | Siemens | | MAGNETOM Trio | 3 | 12-channel | 1900 | 2.26 | 0.5x0.5x1.0 |
|  | 2 | X | Siemens | | MAGNETOM Trio | 3 | 12-channel | 1900 | 2.26 | 0.5x0.5x1.0 |
|  | 3 | X | Siemens | | MAGNETOM Trio | 3 | 32-channel | 1900 | 2.26 | 0.5x0.5x1.0 |
|  | 4 | X | Siemens | | MAGNETOM Trio | 3 | 32-channel | 1900 | 2.26 | 0.5x0.5x1.0 |
| 2 | 1 | Y | Siemens | | MAGNETOM Trio | 3 | 12-channel | 2300 | 2.93 | 1.1x1.1x1.1 |
|  | 2 | Z | Siemens | | MAGNETOM Trio | 3 | 12-channel | 2300 | 2.93 | 1.1x1.1x1.1 |
|  | 3 | Z | Siemens | | MAGNETOM Trio | 3 | 12-channel | 2300 | 2.93 | 1.1x1.1x1.1 |
|  | 4 | Z | Siemens | | MAGNETOM Prisma | 3 | 64-channel | 2300 | 2.93 | 1.1x1.1x1.1 |

| **Table S9.** Clinical ratings of the DAWBA according to DSM-IV at age 14. | | |
| --- | --- | --- |
|  | | |
| **DAWBA, clinical rating** | **Frequency** | **Percentage (%)** |
| Separation anxiety disorder | 0 | 0.0 |
| Specific phobias | 2 | 0.6 |
| Social phobia | 0 | 0.0 |
| Panic disorder | 1 | 0.3 |
| Agoraphobia | 0 | 0.0 |
| Post-traumatic stress disorder | 0 | 0.0 |
| Obsessive compulsive disorder | 0 | 0.0 |
| Generalised anxiety disorder | 0 | 0.0 |
| Other Anxiety disorder | 4 | 1.2 |
| Major depression | 6 | 1.9 |
| Other depressive disorder | 7 | 2.2 |
| Undifferentiated anxiety/ depression | 0 | 0.0 |
| Mania/ Bipolar disorders | 0 | 0.0 |
| Selective mutism | 0 | 0.0 |
| Attachment disorder (disinhibition) | 0 | 0.0 |
| Attachment disorder (inhibition) | 0 | 0.0 |
| ADHD combined | 2 | 0.6 |
| ADHD impulsive | 5 | 1.6 |
| ADHD hyperactive | 0 | 0.0 |
| Other hyperkinetik | 0 | 0.0 |
| Oppositional defiant disorder | 1 | 0.3 |
| Conduct disorder | 3 | 0.9 |
| Other disruptive disorder | 0 | 0.0 |
| Other diagnosis | 3 | 0.9 |
| Autism spectrum disorders | 1 | 0.3 |
| Tic disorders, including tourette syndrome | 1 | 0.3 |
| Eating disorders, including anorexia, bulimia and binge eating | 1 | 0.3 |
| Psychosis | 0 | 0.0 |
| Stereotypic movement disorder | 0 | 0.0 |
| Any other disorder | 0 | 0.0 |
|  |  |  |
| ***Note***. n=321, no missing values. Clinical ratings are given by trained raters based on the provisional diagnosis made by the computer algorithm (according to answers tot he structured questions from the computer interviews and questionnaires), summaries of the answers given to the structured questions, and the transcripts of all the answers to open-ended questions. DAWBA = Development and Well-Being Assessment; <https://dawba.info/a0.html>; (Goodman et al., 2000); DSM = Diagnostic and Statistical Manual of Mental Disorders. | | |

| **Table S10.** Results of the SKID Interview according to DSM-IV conducted at age 22 at site 1. | | |
| --- | --- | --- |
|  |  |  |
|  | **Frequency** | **Percentage (%)** |
| **SKID-I** |  |  |
| Bipolar disorder I lifetime | 0 | 0.0 |
| Bipolar disorder I current | 0 | 0.0 |
| Bipolar disorder II lifetime | 0 | 0.0 |
| Bipolar disorder II current | 0 | 0.0 |
| Other bipolar disorder lifetime | 0 | 0.0 |
| Other bipolar disorder current | 0 | 0.0 |
| Major depression lifetime | 12 | 7.5 |
| Major depression current | 3 | 1.9 |
| Dysthymia current | 0 | 0.0 |
| Depressive disorder lifetime | 9 | 5.7 |
| Depressive disorder current | 1 | 0.6 |
| Schizophrenia lifetime | 0 | 0.0 |
| Schizophrenia current | 0 | 0.0 |
| Psychotic disorder lifetime | 1 | 0.6 |
| Psychotic disorder current | 1 | 0.6 |
| Alcohol dependence lifetime | 0 | 0.0 |
| Alcohol dependence current | 0 | 0.0 |
| Any substance dependence lifetime | 3 | 1.9 |
| Any substance dependence current | 1 | 0.6 |
| Panic disorder lifetime | 3 | 1.9 |
| Panic disorder current | 3 | 1.9 |
| Agoraphobia without panic lifetime | 0 | 0.0 |
| Agoraphobia without panic current | 0 | 0.0 |
| Social phobia lifetime | 1 | 0.6 |
| Social phobia current | 2 | 1.3 |
| Specific phobia lifetime | 7 | 4.4 |
| Specific phobia current | 6 | 3.8 |
| Obsessive-compulsive-disorder lifetime | 0 | 0.0 |
| Obsessive-compulsive-disorder current | 0 | 0.0 |
| Posttraumatic stress disorder lifetime | 4 | 2.5 |
| Posttraumatic stress disorder current | 1 | 0.6 |
| Generalized anxiety disorder lifetime | 3 | 1.9 |
| Generalized anxiety disorder current | 2 | 1.3 |
| Somatization disorder current | 0 | 0.0 |
| Pain disorder current | 0 | 0.0 |
| Hypochondriasis current | 0 | 0.0 |
| Body dysmorphic disorder current | 0 | 0.0 |
| Anorexia lifetime | 3 | 1.9 |
| Anorexia current | 0 | 0.0 |
| Bulimia lifetime | 3 | 1.9 |
| Bulimia current | 2 | 1.3 |
| Binge-eating disorder lifetime | 0 | 0.0 |
| Binge-eating disorder current | 0 | 0.0 |
| Adjustment disorder current | 1 | 0.6 |
| Other disorder of DSM-IV, Axis I, lifetime | 1 | 0.6 |
| Other disorder of DSM-IV, Axis I, current | 1 | 0.6 |
| **SKID-II** |  | 0.0 |
| Avoidant personality disorder | 0 | 0.0 |
| Dependent personality disorder | 0 | 0.0 |
| Obsessive-compulsive personality disorder | 0 | 0.0 |
| Negativistic personality disorder | 0 | 0.0 |
| Depressive personality disorder | 1 | 0.6 |
| Paranoid personality disorder | 0 | 0.0 |
| Schizotypal personality disorder | 0 | 0.0 |
| Schizoid personality disorder | 0 | 0.0 |
| Histrionic personality disorder | 0 | 0.0 |
| Narcisstic personality disorder | 0 | 0.0 |
| Borderline personality disorder | 0 | 0.0 |
| Antisocial personality disorder | 1 | 0.6 |
|  |  |  |
| ***Note***. n=159, missings = 19. SKID is a semi-structured interview guide which was administered by trained mental health professional (Wittchen et al., 1997). It followst he multi-axial system: SKID-I for Axis I disorders (major mentla health disorders) and SKID-II for Axis II disorders (personality disorders). SKID = Structured Clinical Interview for DSM; DSM = Diagnostic and Statistical Manual of Mental Disorders. | | |

| **Table S11.** Results of the Mini-International Neuropsychiatric Interview according to DSM-IV conducted at age 22 at site 2. | | |
| --- | --- | --- |
|  |  |  |
|  | **Frequency** | **Percentage (%)** |
| Major depressive episode current | 6 | 3.9 |
| Major depressive episode past | 20 | 13.1 |
| Dysthymia | 3 | 2.0 |
| Hypomania current | 2 | 1.3 |
| Hypomania past | 4 | 2.6 |
| Mania current | 1 | 0.7 |
| Mania past | 0 | 0.0 |
| Panic disorder without agoraphobia current | 4 | 2.6 |
| Panic disorder without agoraphobia lifetime | 8 | 5.2 |
| Panic disorder with agoraphobia current | 2 | 1.3 |
| Agoraphobia without panic | 4 | 2.6 |
| Panic disorder with agoraphobia lifetime | 1 | 0.7 |
| Social phobia | 7 | 4.6 |
| Obsessive-compulsive-disorder | 1 | 0.7 |
| Alcohol dependence current | 3 | 2.0 |
| Substance dependence current | 14 | 9.2 |
| Psychosis lifetime | 3 | 2.0 |
| Mood disorder with psychotic features | 1 | 0.7 |
| Bulimia current | 3 | 2.0 |
| Generalized anxiety disorder | 13 | 8.5 |
| Antisocial personality disorder | 4 | 2.6 |
|  |  |  |
| ***Note***. n=162, missings = 9. The short structured Mini-International Neuropsychiatric Interview (Sheehan et al., 1998) was administered by trained mental health professional. DSM = Diagnostic and Statistical Manual of Mental Disorders. | | |

# Supplemental Figures

| **Figure S1.** Age and Sex Distribution |
| --- |
|  |
| 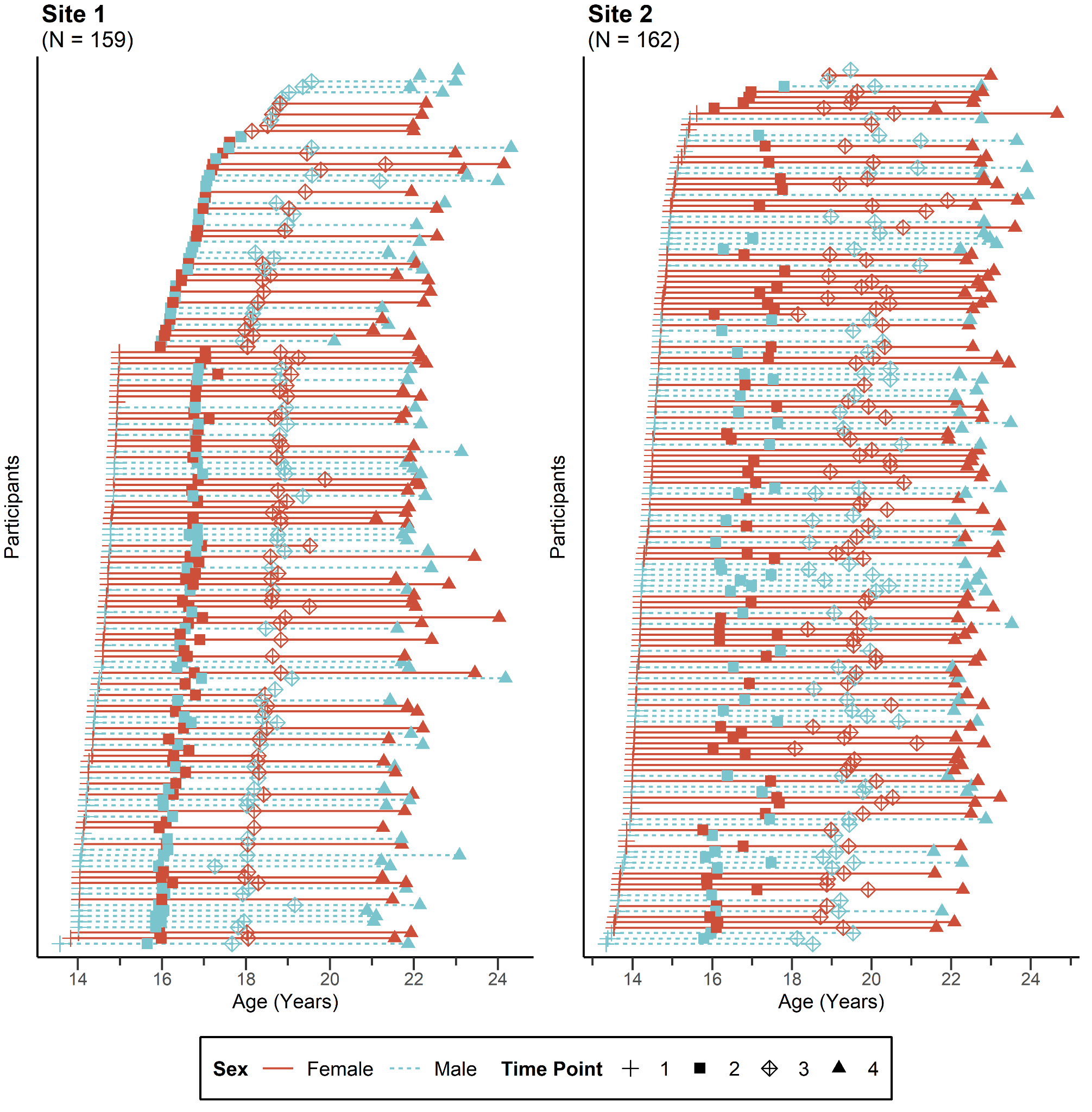 |

| **Figure S2.** Distributions of Negative Life Events, Emotional Symptoms and Depressive Symptoms |
| --- |
|  |
| 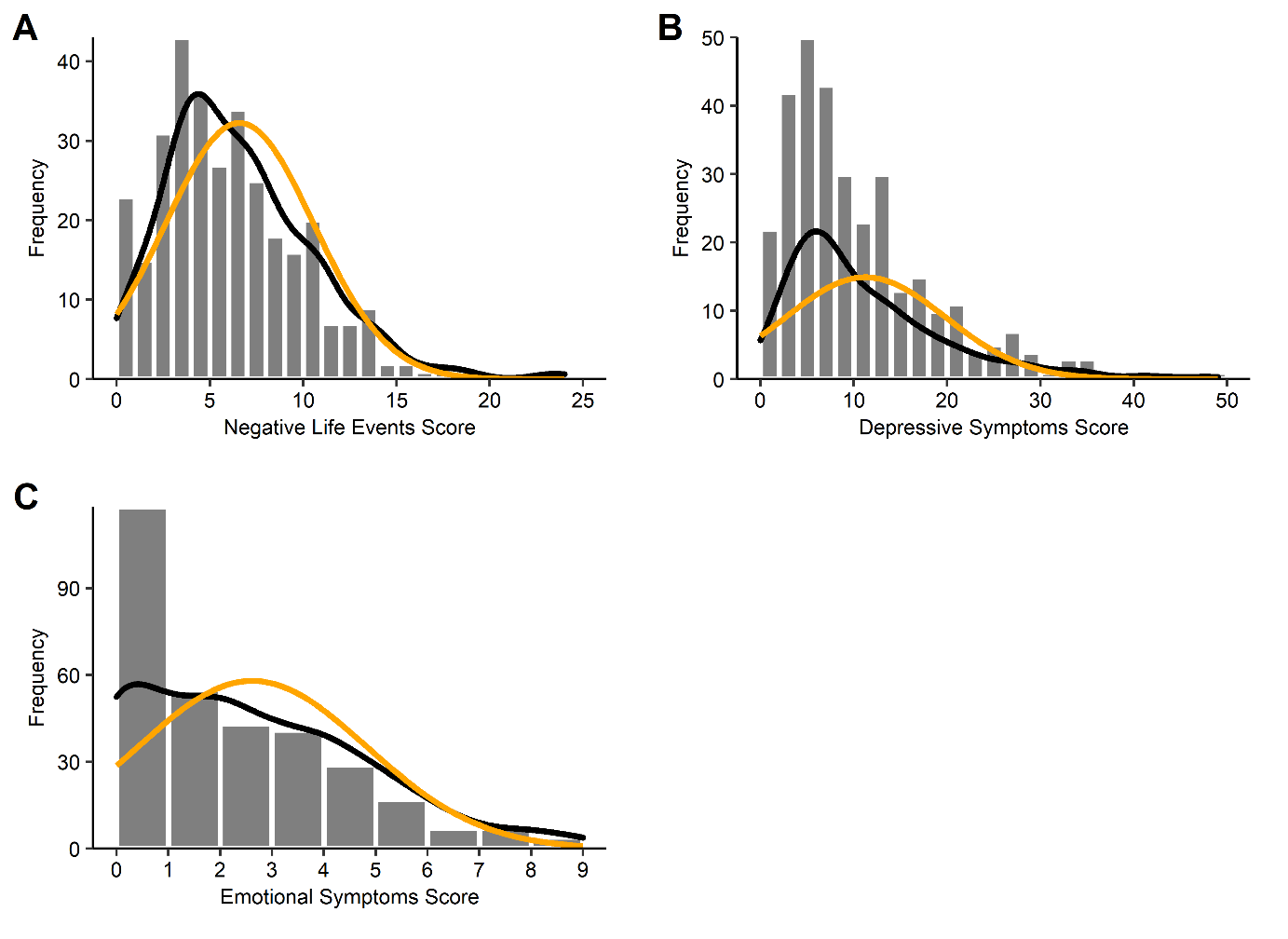 |
|  |
| ***Note.*** Histograms and density plots of negative life events and emotional symptoms at the first time point and depressive symptoms at the fourth time point. The black line represents the observed densities and the orange line shows the normal distribution curve. Negative life events score was measured with the Life events questionnaire (LEQ; Newcomb et al., 1981), depressive symptoms score with the Center for Epidemiologic Studies Depression Scale (CES-D; Radloff, 1977), and emotional symptoms score via the Strengths and Difficulties Questionnaire (SDQ; Goodman, 1997). |

| **Figure S3.** Distribution of Orbitofrontal Cortical Thickness per Time Point |
| --- |
|  |
| 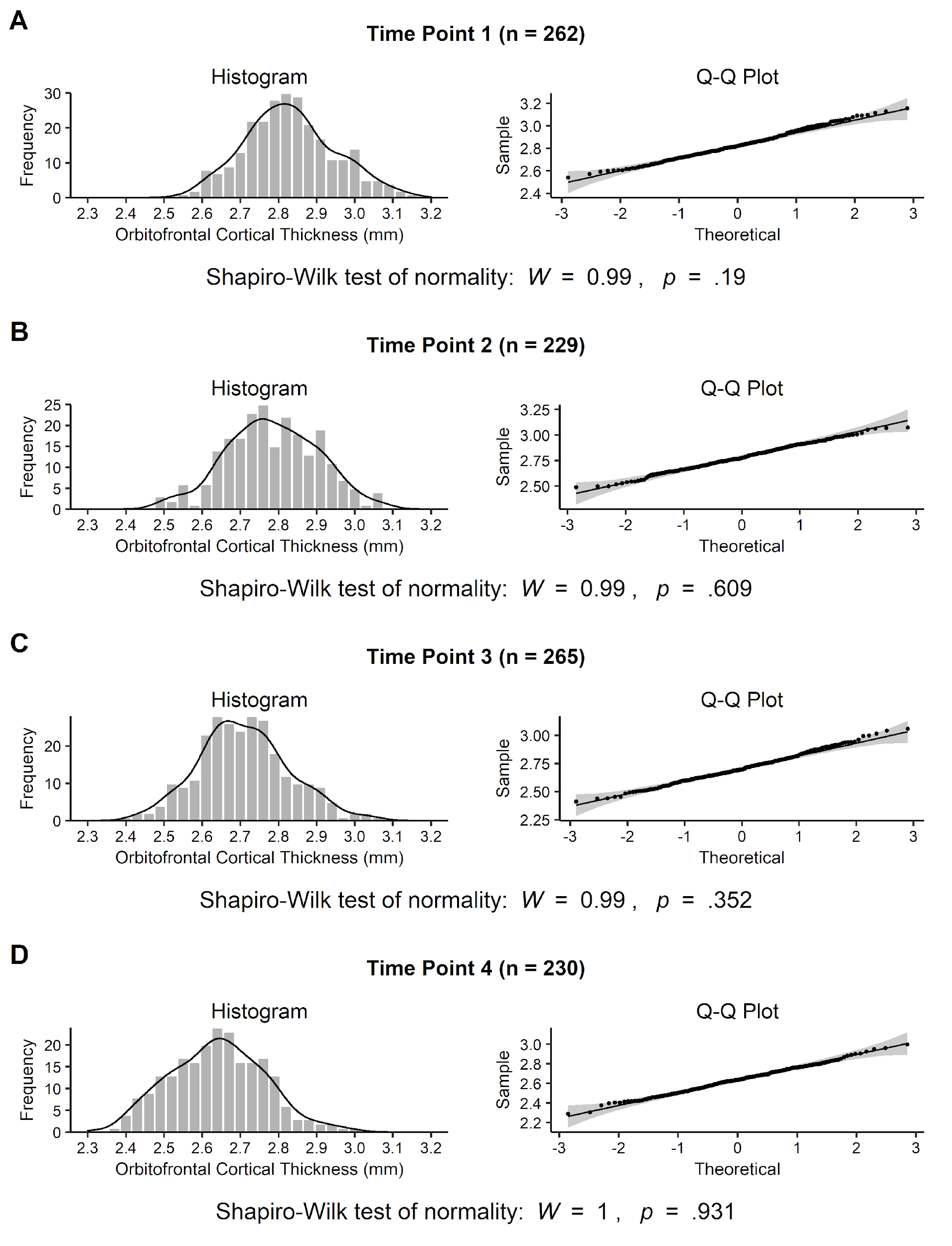 |
|  |
| ***Note.*** Distribution and test of normality of orbitofrontal cortical thickness per time point in the pooled sample. |

| **Figure S4.** Missing Data Patterns |
| --- |
|  |
| 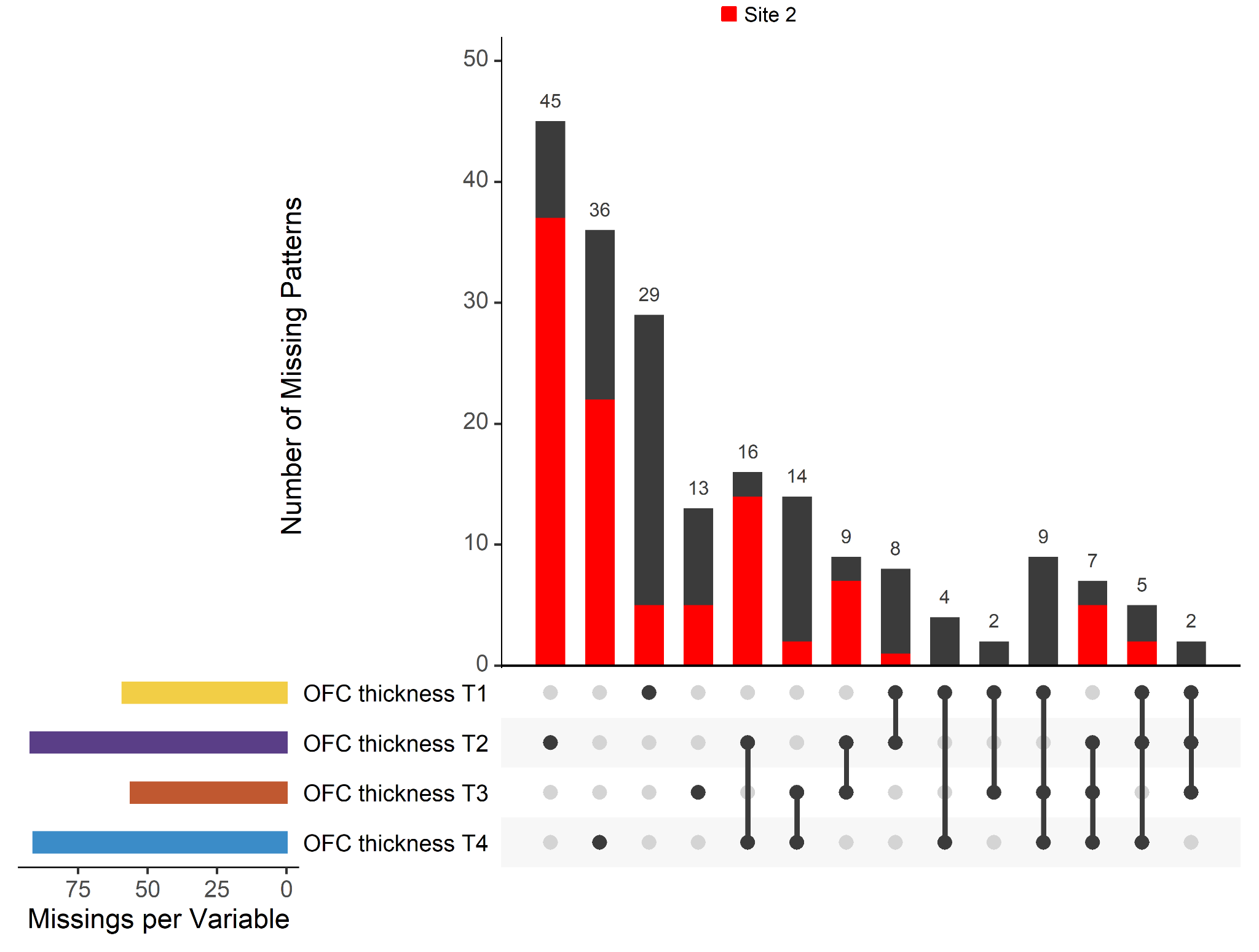 |
|  |
| ***Note.*** Of the variables of interest in the final multiple-mediators model, only the ones shown had missings. The horizontal bar chart shows the missings per set (variable). The missing data patterns are visualized at the bottom right by highlighted and connected sets (intersections). The vertical bar plot shows the frequency of these missing data patterns with the proportion of missing patterns in the subsample from Site 2. |

| **Figure S5.** Evaluation of Model Fit: Sample and Estimated Means and Proportions |
| --- |
|  |
| 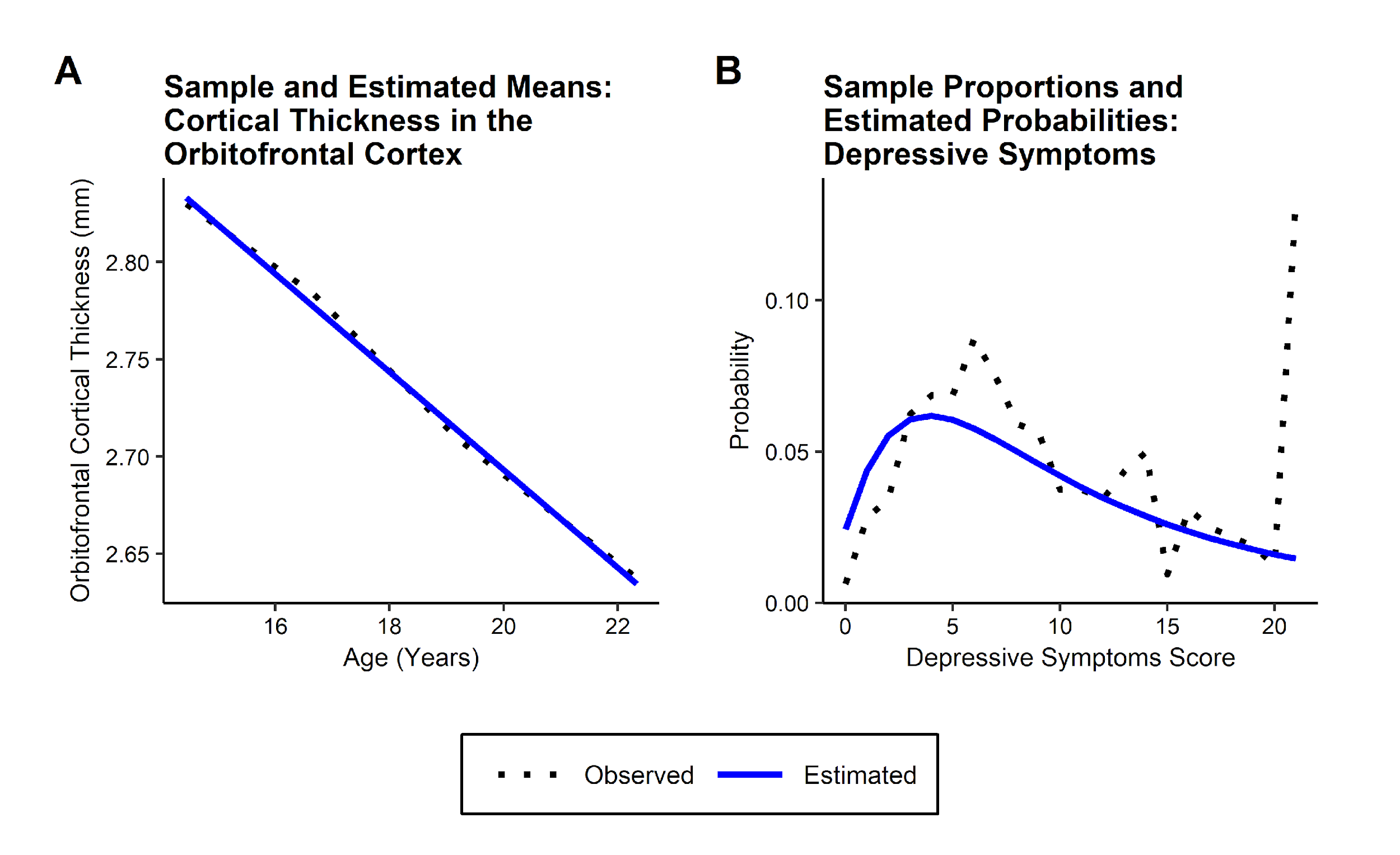 |
|  |
| ***Note.*** Graphical evaluation of the extent to which the multiple-mediators model is accurately recovering the observed sample means and proportions. A: The solid blue line shows the estimated course of orbitofrontal cortical thickness; the dotted black line connects the mean observed cortical thickness at the four time points. B: The solid blue line shows the estimated probability of depressive symptoms under the assumption of a negative binomial distribution; the dotted black line depicts the observed proportion of depressive symptoms. The depressive symptoms score was measured with the Center for Epidemiologic Studies Depression Scale (CES-D; Radloff, 1977). |

| **Figure S6.** Effects of Emotional Symptoms and Negative Life Events on Orbitofrontal Cortical Thickness |
| --- |
|  |
| 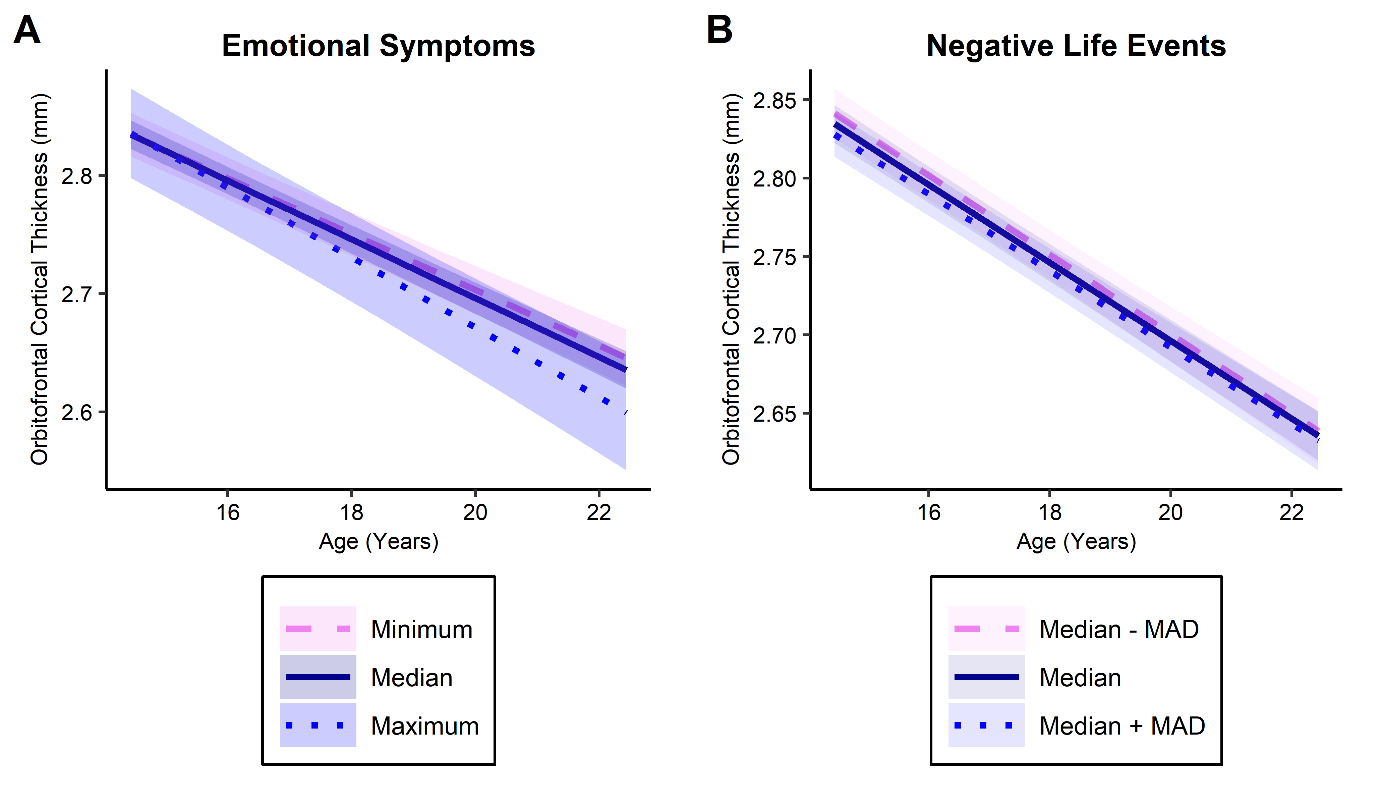 |
|  |
| ***Note.*** Effects are probed based on the results of the multiple-mediators model. All covariates except the one plotted are held constant at their mean or median. Emotional symptoms were measured at the first time point with the Strengths and Difficulties Questionnaire emotion symptoms score (SDQ; Goodman, 1997). Negative Life Events before the first time point were measured with the Life Events Questionnaire (LEQ; Newcomb et al., 1981). |

| **Figure S7.** Effects of Baseline Emotional Symptoms on Depressive Symptoms in Early Adulthood |
| --- |
|  |
| 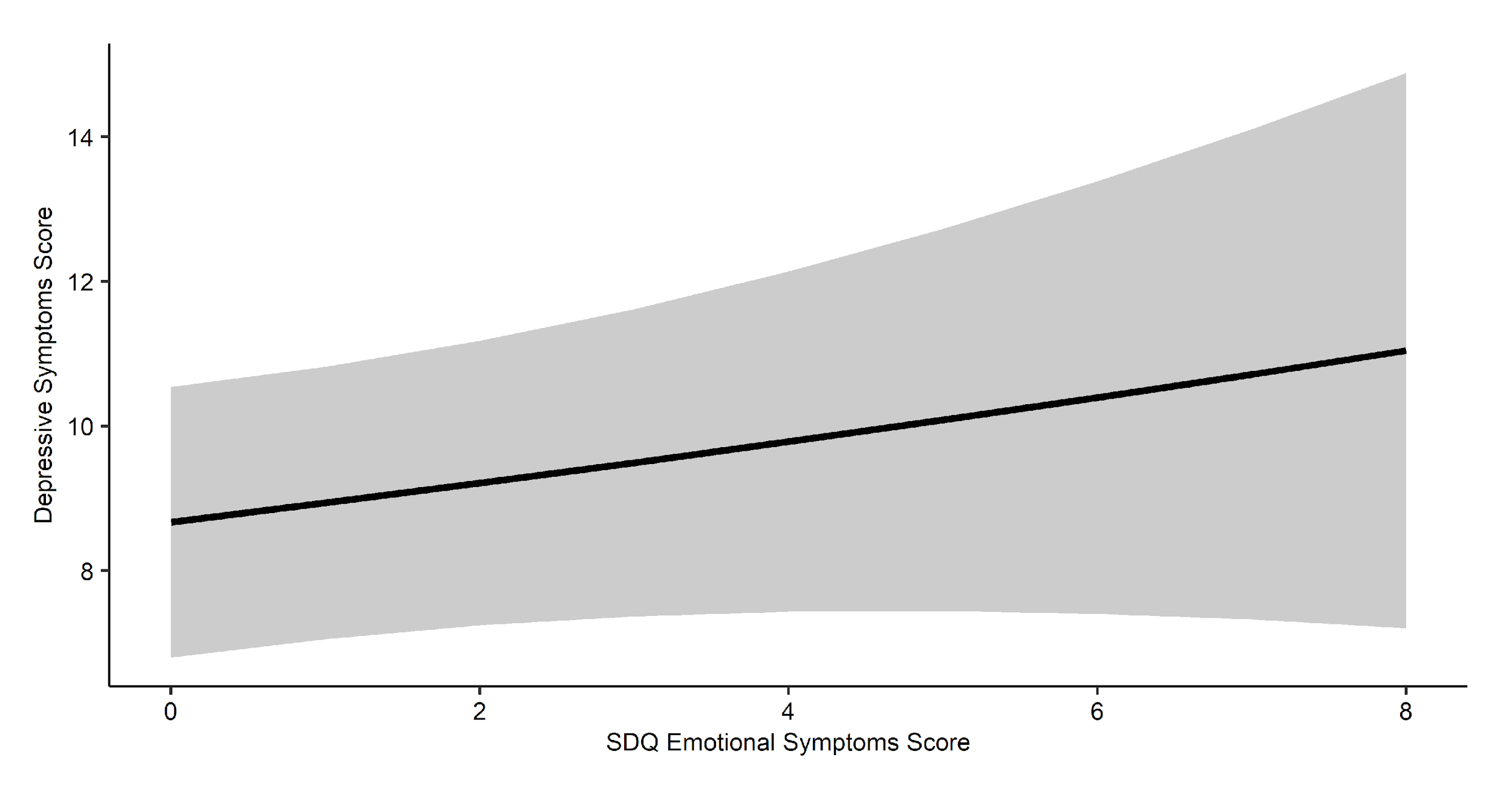 |
|  |
| ***Note.*** The Effect is probed based on the results of the multiple-mediators model. All covariates except emotional symptoms are held constant at their mean or median. The Strengths and Difficulties emotional symptoms score (SDQ; Goodman, 1997) was assessed as a proxy for baseline depressive symptoms. Depressive symptoms score in early adulthood was measured with the Center for Epidemiologic Studies Depression Scale (CES-D; Radloff, 1977) during the fourth time point. |

# Supplemental References

Goodman, R. (1997). The Strengths and Difficulties Questionnaire: A Research Note. *Journal of Child Psychology and Psychiatry*, *38*(5), 581–586. https://doi.org/10.1111/j.1469-7610.1997.tb01545.x

Newcomb, M. D., Huba, G. J., & Bentler, P. M. (1981). A Multidimensional Assessment of Stressful Life Events among Adolescents: Derivation and Correlates. *Journal of Health and Social Behavior*, *22*(4), 400–415. JSTOR. https://doi.org/10.2307/2136681

Petersen, A. C., Crockett, L., Richards, M., & Boxer, A. (1988). A self-report measure of pubertal status: Reliability, validity, and initial norms. *Journal of Youth and Adolescence*, *17*(2), 117–133. https://doi.org/10.1007/BF01537962

Radloff, L. S. (1977). The CES-D Scale: A Self-Report Depression Scale for Research in the General Population. *Applied Psychological Measurement*, *1*(3), 385–401. https://doi.org/10.1177/014662167700100306

Saunders, J. B., Aasland, O. G., Babor, T. F., de la Fuente, J. R., & Grant, M. (1993). Development of the Alcohol Use Disorders Identification Test (AUDIT): WHO Collaborative Project on Early Detection of Persons with Harmful Alcohol Consumption—II. *Addiction (Abingdon, England)*, *88*(6), 791–804. https://doi.org/10.1111/j.1360-0443.1993.tb02093.x

Wechsler, D. (2003). *Wechsler Intelligence Scale for Children, 4th Edn*. San Antonia, TX: PsychCorp.
